# Immunoglobulin signature predicts risk of post-acute COVID-19 syndrome

**DOI:** 10.1038/s41467-021-27797-1

**Published:** 2022-01-25

**Authors:** Carlo Cervia, Yves Zurbuchen, Patrick Taeschler, Tala Ballouz, Dominik Menges, Sara Hasler, Sarah Adamo, Miro E. Raeber, Esther Bächli, Alain Rudiger, Melina Stüssi-Helbling, Lars C. Huber, Jakob Nilsson, Ulrike Held, Milo A. Puhan, Onur Boyman

**Affiliations:** 1 Department of Immunology, University Hospital Zurich, University of Zurich, Zurich, Switzerland; 2 Epidemiology, Biostatistics and Prevention Institute, University of Zurich, Zurich, Switzerland; 3 Clinic for Internal Medicine, Uster Hospital, Uster, Switzerland; 4 Department of Medicine, Limmattal Hospital, Schlieren, Switzerland; 5 Clinic for Internal Medicine, City Hospital Triemli Zurich, Zurich, Switzerland; 6 Faculty of Medicine, University of Zurich, Zurich, Switzerland

**Keywords:** Predictive markers, Infection, Risk factors

## Abstract

Following acute infection with severe acute respiratory syndrome coronavirus 2 (SARS-CoV-2) a significant proportion of individuals develop prolonged symptoms, a serious condition termed post-acute coronavirus disease 2019 (COVID-19) syndrome (PACS) or long COVID. Predictors of PACS are needed. In a prospective multicentric cohort study of 215 individuals, we study COVID-19 patients during primary infection and up to one year later, compared to healthy subjects. We discover an immunoglobulin (Ig) signature, based on total IgM and IgG3 levels, which – combined with age, history of asthma bronchiale, and five symptoms during primary infection – is able to predict the risk of PACS independently of timepoint of blood sampling. We validate the score in an independent cohort of 395 individuals with COVID-19. Our results highlight the benefit of measuring Igs for the early identification of patients at high risk for PACS, which facilitates the study of targeted treatment and pathomechanisms of PACS.

## Abstract (web summary)

Studying a prospective cohort, the authors develop and validate a predictive score for post-acute COVID-19 syndrome, also known as long-COVID. This score relies on an immunoglobulin signature and is independent of timepoint of blood sampling.

## Introduction

Infection with severe acute respiratory syndrome coronavirus 2 (SARS-CoV-2) can cause asymptomatic or symptomatic coronavirus disease 2019 (COVID-19). As of October 25, 2021, more than 244 million SARS-CoV-2 infections have been confirmed worldwide that have caused at least 5 million deaths. Symptoms of acute SARS-CoV-2 infection can include fever, fatigue, myalgia, weakness, headache, rhinorrhea, dry cough, shortness of breath (dyspnea), change in smell or taste, nausea, vomiting, and diarrhea^1^. Following infection, a rapid systemic immune response is mounted against SARS-CoV-2, characterized by increased serum concentrations of chemokines and pro-inflammatory cytokines, such as interleukin (IL)-6 and tumor necrosis factor (TNF), and the appearance of activated monocytes, followed by SARS-CoV-2-specific immunoglobulin M (IgM), IgA, and IgG antibodies and interferon-γ-producing T cells^2–7^. This concerted action of the immune system controls the replication of SARS-CoV-2, and infectious SARS-CoV-2 cannot be isolated from the respiratory tract after 3 weeks^8^. This typically coincides with the recovery of most individuals with symptomatic COVID-19.

However, about one-third of individuals report one or more COVID-19-related symptoms that last for more than 4 weeks (i.e. 29 days and more) after the onset of the first COVID-19-related symptom^9,10^, a condition termed post-acute COVID-19 syndrome (PACS) or long COVID. Community prevalence of PACS has been estimated in most studies to lie between 10% and 60%, which depends on the definition of PACS used and patient care level^10,11^. PACS can be further subdivided into subacute COVID-19 when COVID-19-related symptoms last 12 weeks (84 days) or less versus post-COVID-19 syndrome, which defines patients with COVID-19-related symptoms persisting for more than 84 days after onset of their first symptoms of COVID-19^12^. The most frequent symptoms of PACS are reported to be fatigue, dyspnea, and cognitive impairment (also termed “brain fog”, which includes loss of concentration and memory), as well as pain and aches at different sites (including headache), cough, change in smell or taste, and diarrhea^12,13^. As PACS is increasingly recognized as a serious consequence of SARS-CoV-2 infection, early identification of individuals at risk of developing PACS is needed.

A recent study analyzed PACS in individuals who self-reported their symptoms by using an app. The authors found PACS to correlate with increased hospitalization rate and comorbidities, such as lung disease, asthma bronchiale, and heart disease, and they concluded that age, female sex, and number of symptoms during the first week of disease could be used to estimate an individual’s risk for PACS^14^. However, self-reported data and telehealth are at risk for bias, and risk factors associated with a severe course of primary SARS-CoV-2 infection complicate the detection of underlying risk factors for PACS independent of disease severity. To address these issues, we have characterized a prospective cohort of 215 individuals by clinical visits and laboratory analyses up to one year of follow-up. We found distinct patterns of total immunoglobulin (Ig) levels in patients with COVID-19 and integrated these in a clinical prediction score, which allowed early identification of both outpatients and hospitalized individuals with COVID-19 that were at high risk for PACS.

## Results

### Characteristics of COVID-19 patients with and without PACS

**Fig. 1:**
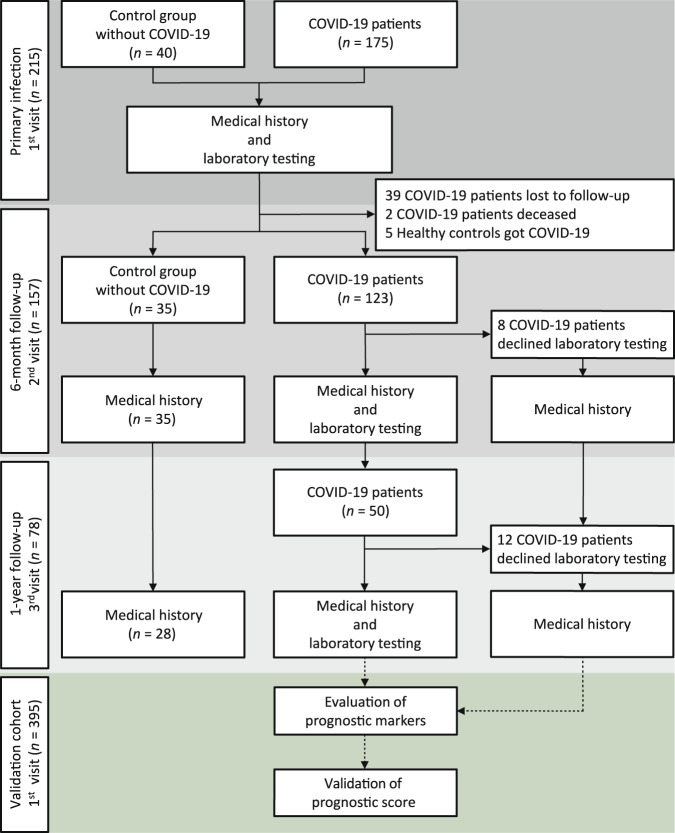
Flow chart of COVID-19 patients and healthy controls enrolled in the study. Flow chart of individuals with confirmed SARS-CoV-2 infection (COVID-19 patients; *n* = 175) and healthy controls (Control group; *n* = 40) with no history of COVID-19-related symptoms, a negative SARS-CoV-2 S1-specific immunoassay, no history of autoimmune disease, and no active illness prior to blood sampling. Medical history and a blood sample were obtained at the first visit (*n* = 175), corresponding to primary SARS-CoV-2 infection in COVID-19 patients, second visit (*n* = 123) at about 6 months after primary infection (6-month follow-up), and third visit (*n* = 50) at about one year after primary infection. At 6-month follow-up, *n* = 39 patients declined follow-up or were unreachable, *n* = 2 patients deceased, and *n* = 5 healthy controls got COVID-19. At 6-month follow-up *n* = 8 and at 1-year follow-up *n* = 12 patients only declined laboratory testing. *n* = 134 patients were followed-up at least once, including 11 patients that only attended a 1-year follow-up. Healthy controls were clinically followed-up after 6 months (*n* = 35) and 1 year (*n* = 28). Data were validated in a separate validation cohort of *n* = 395 individuals with confirmed COVID-19 that were followed up for 6 months.

Our multicentric cohort included 175 individuals with reverse-transcriptase quantitative polymerase chain reaction-confirmed SARS-CoV-2 infection as well as 40 healthy controls without acute symptoms and negative SARS-CoV-2-specific immunoassays. A total of 134 individuals were followed up, including 123 patients at about 6 months and 50 patients at one year after primary SARS-CoV-2 infection (Fig. 1). Based on the classification by the World Health Organization^15^, we distinguished 89 mild and 45 severe COVID-19 cases attending follow-up and further subclassified them into four cases of asymptomatic disease, 76 mild illness, nine mild pneumonia, 20 severe pneumonia, and 25 acute respiratory distress syndrome (ARDS), including five mild, 10 moderate, and 10 severe ARDS cases (Table 1).

**Table 1:** Participant characteristics at inclusion and 6-month follow-up.

Participant characteristics	Controls (*n* = 40)	Mild COVID-19 cases (*n* = 89)				Severe COVID-19 cases (*n* = 45)					Validation cohort (*n* = 395)	
Disease grade^a^	Healthy	Asymptomatic	Mild illness		Mild pneumonia	Severe pneumonia	Mild ARDS		Moderate ARDS	Severe ARDS	Mild COVID-19	Severe COVID-19
Disease grade—no.	40	4	76		9	20	5		10	10	386	9
Age—median (IQR) [yrs]	33 (29–53)	34 (27–49)				64 (54–74)					51 (34–67)	
Sex—male no. (%)	17 (42.5)	43 (48.3)				32 (71.1)					199 (50.4)	
Level of care^b^—hospitalized no. (%)	–	12 (13.5)				45 (100)					17 (4.3)	
No. of symptoms^c^ (IQR)	–	2 (1–3)				3 (2–3)					2 (1–3)	
*Pre-existing comorbidities and treatments*—no. (%)												
Cardiovascular disease	1 (2.5)	1 (1.1)				17 (37.8)					20 (5.1)	
Diabetes mellitus	1 (2.5)	4 (4.5)				15 (33.3)					7 (1.8)	
Hypertension	5 (12.5)	7 (7.9)				24 (53.3)					54 (13.7)	
Kidney disease	0	3 (3.4)				12 (26.7)					2 (0.5)	
Lung disease	6 (15.0)	9 (10.1)				12 (26.7)					29 (7.3)	
Asthma bronchiale^d^	5 (12.5)	9 (10.1)				8 (17.8)					12 (3.1)	
Malignancy	1 (2.5)	3 (3.4)				5 (11.1)					22 (5.6)	
Body-Mass-Index—median (IQR)^d^	23 (21–25)	25 (22–26)				28 (25–31)					24 (22–26)	
Systemic immunosuppression	0	0				9 (20.0)					10 (2.5)	
*Clinical follow-up*^e^												
Any prolonged symptom—no. (%)	3 (8.6)	48 (53.9)				37 (82.2)					216 (54.7)	
Anxiety, depression—no. (%)	1 (2.9)	7 (7.9)				13 (28.9)					120 (30.4)	
—median (IQR) [d]^f^	90	134 (89–179)				151 (100–229)						
Chest pain—no. (%)	0	6 (6.7)				6 (13.3)					22 (5.6)	
—Median (IQR) [d]	–	189 (125–225)				324 (221–384)						
Cough—no. (%)	0	2 (2.2)				8 (17.8)					65 (16.5)	
——median (IQR) [d]	–	142 (87–196)				67 (45–85)						
Dyspnea—no. (%)	0	6 (6.7)				20 (44.4)					47 (11.9)	
—median (IQR) [d]	–	215 (90–246)				205 (115–361)						
Fatigue—no. (%)	1 (2.9)	23 (25.8)				23 (51.1)					94 (23.8)	
—median (IQR) [d]	40	156 (42–206)				292 (213–369)						
GIT symptoms—no. (%)	0	3 (3.4)				4 (8.9)					42 (10.7)	
—median (IQR) [d]	–	239 (189–245)				229 (226–271)						
Headache—no. (%)	1 (2.9)	5 (5.6)				1 (2.2)					61 (15.4)	
—median (IQR) [d]	40	156 (108–211)				194						
Smell/taste disorder—no. (%)	0	18 (20.2)				8 (17.8)					50 (12.7)	
—median (IQR) [d]	–	128 (43–199)				158 (102–190)						
Other symptoms^g^—no. (%)	0	6 (6.7)				8 (17.8)					184 (46.6)	
New or aggravated comorbidity	1 (2.9)	7 (7.9)				13 (28.9)					–	
*Laboratory characteristics*—median (IQR)												
**Timepoint of blood sampling**	**–**	**Primary infection**		**6-month follow-up**^h^		**Primary infection**		**6-month follow-up**^h^			**Primary infection**	
Time after symptom onset [days]	**–**	9 (7–16)		195 (185–206)		14 (9–28)		211 (194–225)			19 (17–22)	
CRP [mg/L]	0.6 (0.4–1.6)	1.2 (0.5–4.3)		0.6 (0.6–1.2)		60.5 (27.0–120.0)		2.2 (1.7–5.1)			–	
IL-6 [pg/mL]	0.5 (0.0–1.1)	1.1 (0.0–3.9)		1.1 (0.5–2.3)		20.7 (8.4–60.5)		1.7 (0.3–5.4)			–	
TNF [pg/mL]	8.1 (6.4–10.0)	9.8 (7.7–11.6)		9.2 (6.8–11.4)		16.5 (13.8–20.5)		12.0 (9.5–15.2)			–	
Leukocytes [10^9^/L]	6.1 (5.1–7.2)	5.5 (4.5–6.8)		6.4 (5.5–7.0)		6.4 (5.0–9.1)		6.9 (5.5–8.3)			–	
Neutrophils [10^9^/L]	3.4 (2.8–4.4)	3.1 (2.4–4.0)		3.4 (2.7–3.9)		4.7 (3.4–7.2)		4.2 (3.2–5.1)			–	
Lymphocytes [10^9^/L]	1.8 (1.5–2.3)	1.9 (1.4–2.2)		2.0 (1.7–2.5)		0.7 (0.6–1.2)		1.8 (1.5–2.4)			–	
NLR	1.8 (1.4–2.3)	1.6 (1.2–2.2)		1.6 (1.3–2.0)		5.3 (3.4–11.1)		2.4 (1.5–3.3)			–	
SARS-CoV-2 IgA [OD ratio]	0.3 (0.2–0.5)	1.7 (0.7–4.7)		2.2 (1.5–3.7)		7.3 (2.5–11.3)		5.2 (3.1–7.9)			–	
SARS-CoV-2 IgG [OD ratio]	0.2 (0.2–0.2)	0.6 (0.3–2.0)		2.4 (1.2–4.1)		4.6 (0.6–9.9)		7.1 (5.3–8.6)			–	
Total IgM [g/L]	1.1 (0.8–1.6)	1.1 (0.9–1.5)		1.0 (0.7–1.4)		0.8 (0.6–1.2)		0.7 (0.5–0.9)			0.8 (0.5–1.2)	
Total IgA [g/L]	1.8 (1.5–2.3)	2.1 (1.7–2.8)		2.2 (1.7–2.8)		2.6 (2.0–3.4)		2.4 (2.0–3.2)			1.6 (1.2–2.2)	
Total IgG [g/L]	10.8 (9.5–11.9)	11.4 (10.1–13.0)		11.2 (10.0–12.9)		10.3 (8.3–12.9)		11.5 (9.9–13.1)			8.7 (7.4–10.2)	
Total IgG1 [g/L]	5.4 (4.5–6.2)	6.0 (4.6–6.8)		5.6 (4.4–6.6)		5.1 (4.2–7.0)		5.7 (4.5–6.8)			4.2 (3.3–5.0)	
Total IgG2 [g/L	3.7 (3.0–4.4)	4.1 (3.1–4.8)		3.8 (3.0–4.6)		3.1 (2.5–4.1)		3.2 (2.6–4.4)			2.7 (1.9–3.3)	
Total IgG3 [g/L]	0.5 (0.4–0.7)	0.7 (0.5–1.0)		0.6 (0.5–0.9)		0.7 (0.5–1.2)		0.7 (0.5–1.0)			0.5 (0.3–0.7)	
Total IgG4 [g/L]	0.3 (0.2–0.5)	0.3 (0.2–0.5)		0.3 (0.2–0.5)		0.3 (0.2–0.5)		0.3 (0.2–0.5)			0.2 (0.1–0.4)	

*ARDS* acute respiratory distress syndrome, *CRP* C-reactive protein, *d* days, *GIT* gastrointestinal tract, *Ig* immunoglobulin, *IL* interleukin, *IQR* interquartile range, *NRL* neutrophil-to-lymphocyte ratio, *OD* optical density, *TNF* tumor necrosis factor.

^a^COVID-19 grade according to World Health Organization guidelines was prospectively followed until recovery. Asymptomatic disease, mild illness, and mild pneumonia are considered mild COVID-19 and severe pneumonia, as well as ARDS, are considered severe COVID-19.

^b^Level of care was prospectively followed until recovery.

^c^Five symptoms were systematically recorded during primary infection (fever, fatigue, cough, dyspnea, and gastrointestinal symptoms).

^d^Missing information on asthma bronchiale of 6 patients in validation cohort, and on Body-Mass-Index of 23 patients in derivation cohort as well as 6 healthy controls.

^e^Listed symptoms were reported to have endured longer than four weeks after symptom onset and could not be explained by another disease than COVID-19 or any disease in the control group. Clinical follow-up of healthy controls includes only individuals that did not get COVID-19 during follow-up (*n* = 35). Nobody reported prolonged fever.

^f^Median duration of prolonged symptoms only considers the duration of symptoms that were prolonged and does not represent the median duration of symptoms in all COVID-19 patients.

^g^Other reported symptoms were not assessed systematically and include cognitive impairment, peripheral neuropathy, skin rash, palpitation, joint and muscle pain. All reported symptoms to adhere to the NICE guidelines^12^. For the validation cohort, other reported symptoms include sore throat, difficulty swallowing or lump in the throat, nasal congestion, sneezing, swollen lymph nodes, myalgia, arthralgia, sleep disturbances, concentration difficulties, memory difficulties, vertigo, palpitations, hair loss, skin rash, tingling of upper or lower extremities, tremors, irritated eyes, visual disturbances, and hearing disturbances.

^h^6-month follow-up: Mild COVID-19 cases (*n* = 77), severe COVID-19 cases (*n* = 38).

53.9% of mild COVID-19 cases and 82.2% of patients that developed severe COVID-19 had PACS, defined—as aforementioned—by the persistence of one or more COVID-19-related symptoms for more than four weeks (i.e. 29 days and more) after the onset of the first COVID-19-related symptom (Table 1). Conversely, only 8.6% of healthy controls experienced one or more symptoms for more than 28 days during the one-year follow-up period (Table 1). The most common prolonged symptoms were fatigue, dyspnea, a change in smell or taste, and anxiety or depression. Symptoms of PACS were about 2–6.5-fold more frequent in severe compared to mild COVID-19 cases overall, with the exception of smell or taste disorders (Table 1).

In patients with severe disease, laboratory values taken at primary infection showed signs of lymphopenia and systemic inflammation, including increased concentrations of C-reactive protein (CRP), IL-6, and TNF, and some of these inflammatory markers remained perturbed at 6-month follow-up (Table 1).

When studying the above-mentioned demographic characteristics, comorbidities, and laboratory values at primary SARS-CoV-2 infection in individuals experiencing PACS, we observed several differences (Table 2). Compared to individuals without PACS, the group of patients experiencing PACS contained a larger percentage of severe COVID-19 cases (odds ratio 3.87; *p* = 0.001), showed more COVID-19-related symptoms during primary infection (odds ratio 1.81; *p* = 0.001), were of higher age (odds ratio 1.67; *p* = 0.008), and more often required hospitalization (odds ratio 2.55; *p* = 0.014) (Table 2). Sex distribution between the groups of our cohort with and without PACS was similar (*p* = 0.840). Moreover, we observed an association of risk of developing PACS with a history of lung disease (odds ratio 6.29; *p* = 0.004) and, particularly, asthma bronchiale (odds ratio 9.74; *p* = 0.003) (Table 2). Furthermore, CRP and TNF concentrations were slightly higher at primary SARS-CoV-2 infection in individuals later developing PACS, although the inflammatory parameters did not have largely increased odds ratios (odds ratios 1.01 and 1.07; *p* = 0.022 and 0.049, respectively) (Table 2). Collectively, we observed that several determinants of severe COVID-19, including age, hospitalization, and an increase of certain inflammatory markers, present during primary infection correlated with an increased risk of developing PACS.

**Table 2:** Characteristics of patients during primary SARS-CoV-2 infection correlating with post-acute COVID-19 syndrome (PACS).

COVID-19 cases	Without PACS (*n* = 49)	With PACS (*n* = 85)		Odds ratio (CI)^*a*^	*p* value
Symptom duration categories	Acute COVID-19 (<4 weeks)	Subacute COVID-19 (>4 weeks)	Post-COVID-19 syndrome (>12 weeks)	–	–
Symptom duration—no.	49	24	61	–	–
Severe COVID-19 cases—no. (%)	8 (16.3)	7 (29.2)	30 (49.2)	3.87 (1.67–9.89)	0.001
*Demographics*^b^					
Age—median (IQR)	34 (27–50)	52 (34–65)		1.67 (1.15–2.49)	0.008
Sex—male no. (%)	28 (57.1)	47 (55.3)		0.93 (0.45–1.89)	0.840
Level of care^c^—hospitalized no. (%)	13 (26.5)	41 (48.2)		2.55 (1.20–5.65)	0.014
Days hospitalized—median (IQR)	0 (0–1)	1 (0–16)		1.06 (1.02–1.12)	0.008
No. of symptoms (IQR)^d^	2 (1–3)	3 (2–3)		1.81 (1.31–2.58)	0.001
*Pre-existing comorbidities and treatments*—*no. (%)*					
Hypertension	9 (18.4)	22 (25.9)		1.53 (0.65–3.86)	0.331
Diabetes mellitus	4 (8.2)	15 (17.6)		2.34 (0.78–8.87)	0.135
Cardiovascular disease	6 (12.2)	12 (14.1)		1.16 (0.41–6.32)	0.779
Lung disease	2 (4.1)	19 (22.4)		6.29 (1.70–44.36)	0.004
Asthma bronchiale	1 (2.0)	16 (18.8)		9.74 (1.88–240.26)	0.003
Kidney disease	5 (10.2)	10 (11.8)		1.16 (0.38–4.02)	0.805
History of malignancy	2 (4.1)	6 (7.1)		1.70 (0.36–13.24)	0.525
Systemic immunosuppression	1 (2.0)	8 (9.4)		4.42 (0.76–113.71)	0.109
Body-Mass-Index—median (IQR)^e^	25 (22–27)	26 (23–29)		1.07 (0.99–1.18)	0.116
*Laboratory characteristics during primary infection*—median (IQR)					
Timepoint of first sampling [days]^f^	9 (6–15)	12 (7–19)		1.05 (1.01–1.10)	0.022
CRP (mg/L)	2.4 (0.6–8.8)	11.0 (0.9–60.5)		1.01 (1.00–1.02)	0.022
IL-6 (pg/mL)	2.0 (0.1–9.3)	5.2 (0.8–20.3)		1.01 (1.00–1.02)	0.113
TNF (pg/mL)	10.2 (8.2–13.0)	11.2 (9.1–17.4)		1.07 (1.01–1.15)	0.049
Leukocytes (10^9^/L)	6.0 (5.0–7.0)	5.8 (4.5–7.1)		1.04 (0.95–1.16)	0.472
Neutrophils (10^9^/L)	3.4 (2.6–4.5)	3.5 (2.7–4.4)		1.08 (0.93–1.27)	0.340
Lymphocytes (10^9^/L)	1.8 (1.4–2.2)	1.3 (0.7–2.0)		0.96 (0.77–1.21)	0.711
NLR	1.7 (1.3–2.4)	2.2 (1.6–4.3)		1.01 (0.99–1.06)	0.540
SARS-CoV-2 IgA (OD ratio)	2.5 (0.9–6.1)	3.1 (0.8–8.4)		1.03 (0.99–1.08)	0.218
SARS-CoV-2 IgG (OD ratio)	0.7 (0.3–3.2)	1.1 (0.3–6.3)		1.08 (0.98–1.20)	0.149
Total IgM [g/L]	1.2 (0.9–1.6)	0.9 (0.6–1.3)		0.73 (0.44–1.19)	0.204
Total IgA [g/L]	2.2 (1.7–2.8)	2.4 (1.9–3.1)		1.18 (0.82–1.71)	0.389
Total IgG [g/L]	11.8 (10.3–13.6)	10.9 (9.1–12.8)		0.95 (0.85–1.06)	0.342
Total IgG1 [g/L]	6.1 (4.8–6.9)	5.7 (4.3–6.8)		0.94 (0.79–1.13)	0.522
Total IgG2 [g/L]	4.1 (3.0–5.2)	3.7 (2.8–4.4)		0.84 (0.64–1.09)	0.186
Total IgG3 [g/L]	0.7 (0.5–1.1)	0.6 (0.5–1.0)		0.71 (0.35–1.42)	0.324
Total IgG4 [g/L]	0.3 (0.2–0.6)	0.3 (0.2–0.4)		0.61 (0.24–1.52)	0.284

*CI* confidence interval, *CRP* C-reactive protein, *Ig* immunoglobulin, *IL* interleukin, *IQR* interquartile range, *NRL* neutrophil-to-lymphocyte ratio, *OD* optical density, *PACS* post-acute COVID-19 syndrome, *TNF* tumor necrosis factor, *wks* weeks.

^a^For categorical variables, odds ratios compare the odds of the occurrence of PACS, given the presence of a categorical variable. For continuous variables, odds ratios were calculated using an unadjusted (univariate) logistic regression model predicting the occurrence of PACS.

^b^Demographics during primary infection (1st visit).

^c^Level of care was prospectively followed until recovery.

^d^Five symptoms were systematically recorded during primary infection: fever, fatigue, cough, dyspnea, and gastrointestinal symptoms.

^e^Missing information on Body-Mass-Index of 23 patients.

^f^Days after onset of first COVID-19-related symptoms.

### Distinct immunoglobulin signature correlating with development of PACS

We assessed serum concentrations of IgA and IgG antibodies specific for the SARS-CoV-2 spike protein subunit 1 (S1) and of total Igs. Compared to healthy controls, we detected increased serum titers of SARS-CoV-2 S1-specific IgA and IgG, in both mild and severe COVID-19 cases, with higher titers found in severe COVID-19 cases (Table 1), confirming the previous findings^6^. Comparison of individuals with and without PACS revealed that at primary infection S1-specific IgA and IgG values were similar between these two groups (Table 2).

**Fig. 2:**
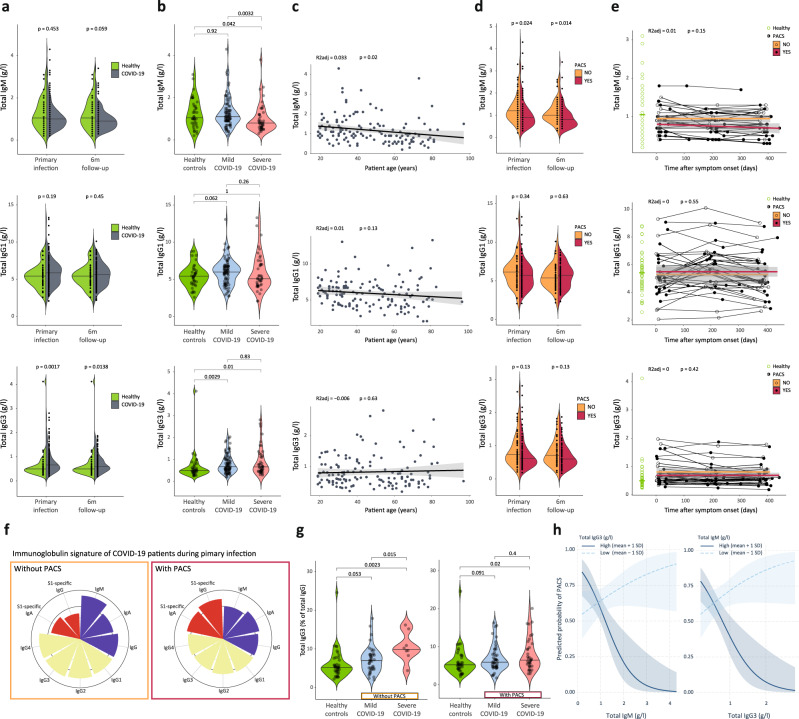
Specific and total immunoglobulins at primary infection and follow-up. **a** and **b** Total serum concentrations of IgM, IgG1, and IgG3 in healthy controls (*n* = 40) versus (**a**) all (*n* = 134 at primary infection; *n* = 115 at 6-month follow-up) or (**b**) mild and severe COVID-19 cases at indicated timepoints (*n* = 89 and 45 respectively). **c** Ig titers at primary infection as a function of age in COVID-19 patients (*n* = 134), with adjusted *R*^2^ (R2adj) and p values of linear models (shown with 95% confidence interval [CI]). **d** Ig signatures in patients without and with PACS, during primary infection (*n* = 49 and 85 respectively) and 6-month follow-up (*n* = 41 and 74 respectively). **e** Ig titers in patients attending all follow-up visits (*n* = 34) as a function of days after symptom onset, with *R*^2^adj and *p* values of generalized additive model (shown with 95% CI). Corresponding patients without (circles) and with PACS (dots) are connected, with a spline visualized for both groups. Green horizontal line indicates median in healthy controls. **f** Radar plots with wedge sizes representing median Ig concentrations of patients without and with PACS (*n* = 49 and 85 respectively), normalized to median concentrations of all patients. **g** IgG3 percentages of total IgG in healthy controls (*n* = 40) and mild and severe COVID-19 cases without (left; *n* = 41 and 8, respectively) and with PACS (right; *n* = 48 and 37, respectively) during primary infection. **h** Interaction plot showing the conditional effects of IgM and IgG3 titers on the predicted probability of PACS in patients with high or low Ig titers (mean ± 1 standard deviation [SD]; *n* = 134, with 85 having PACS), using a logistic regression model (PACS score) adjusted for age, number of symptoms during primary infection, and history of asthma bronchiale (shown with 80% CI for visualization). Variables were compared using a two-sided Wilcoxon’s test.

On measuring total serum concentrations of different Igs, we made several interesting findings. Compared to healthy controls, IgM and IgG1 were indifferent in COVID-19 patients, whereas IgG3 was significantly increased in COVID-19 patients (Fig. 2a). Differentiating mild versus severe COVID-19, IgM was lower in severe compared to mild COVID-19 patients and healthy controls, both at primary infection and 6-month follow-up. IgG1 was indifferent, whereas IgG3 was higher in both mild and severe COVID-19 cases, compared to healthy controls (Fig. 2b and Supplementary Fig. 1a). IgM levels negatively correlated with age, whereas none of the IgG subclasses showed a significant trend with age (Fig. 2c).

In individuals developing PACS, we detected decreased IgM, both at primary infection and 6-month follow-up (Fig. 2d). Whereas IgG1 was unaltered, IgG3 tended to be lower in patients with PACS (Fig. 2d), which was contrary to the increased IgG3 concentrations in both mild and severe COVID-19 cases (Fig. 2a). IgA, IgG2, and IgG4 were neither significantly different in patients with PACS compared to without PACS nor did they show a trend that differed from the one observed in mild and severe COVID-19 cases (Supplementary Fig. 1b–e). Assessment of temporal changes in COVID-19 patients, of whom we had blood samples at primary infection, 6-month, and 1-year follow-up, showed that these total serum Ig concentrations remained stable over time (Fig. 2e and Supplementary Fig. 1f).

In notable contrast to the increased IgG3 concentrations in both mild and severe COVID-19 cases (Fig. 2b), IgG3 showed a trend to being lower in patients developing PACS (Fig. 2d, f). This discrepancy in IgG3 was also evident when analyzing the proportion of IgG3 within total IgG during primary infection, with severe COVID-19 patients without PACS demonstrating increased IgG3, whereas severe COVID-19 patients developing PACS failed to show such increase in IgG3 (Fig. 2g). Other IgG subclasses did not show such changes (Supplementary Fig. 1g).

Notably, individuals with either low IgM or low IgG3 had an increased risk of developing PACS, whereas patients with both high IgM and high IgG3 were less likely to develop PACS (Fig. 2h). In line with this finding, we observed in healthy controls that contracted COVID-19 during the course of this study (Supplementary Table 1), those developing PACS had low IgM prior to SARS-CoV-2 infection, which remained low during the observation period (Supplementary Fig. 1h).

### Building of an immunoglobulin signature-based score predicting PACS

**Fig. 3:**
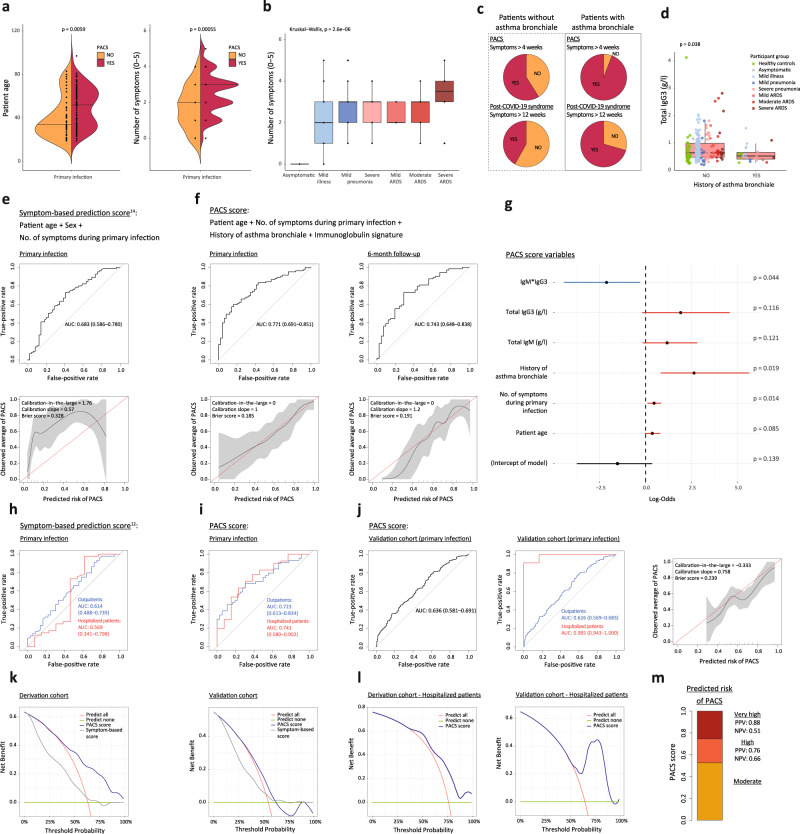
Prediction of post-acute COVID-19 syndrome (PACS) based on clinical features and immunoglobulin signature. **a** Age and number of symptoms during primary infection (0–5; fever, fatigue, cough, dyspnea, gastrointestinal symptoms) in patients without or with PACS. **b** Number of symptoms during primary infection in COVID-19 patients with different disease severities (*n* = 134, with 85 having PACS). **c** PACS and post-COVID-19 syndrome in patients without and with history of asthma bronchiale. **d** IgG3 titers in healthy controls (green symbols) and all COVID-19 patients (*n* = 215; disease severity indicated by colors) at primary infection, without or with history of asthma bronchiale. **e** and **f** Receiver operating characteristic (ROC) curves (top) and calibration plots (bottom) reporting the area under the curve (AUC) with 95% confidence intervals (CI) or calibration-in-the-large, calibration slopes, and Brier scores of logistic regression models for predicting PACS. Use of (**e**) a symptom-based model^14^ and (**f**) the PACS score on data of our patient cohort at primary infection (**e** and **f**, left; *n* = 134, with 85 having PACS) and after shrinkage of coefficients on 6-month follow-up-data (**f**, right; *n* = 115, with 74 having PACS). **g** Regression coefficients of PACS score with 95% CI and *p* values. **h** and **i** ROC curves reporting AUC with CI of PACS score in outpatients (blue) and hospitalized patients (red) of derivation cohort (*n* = 80 and 54, with 44 and 41 having PACS, respectively). **j** Validation of PACS score in independent cohort at primary infection (*n* = 389, with 212 having PACS) and subgroup analysis in outpatients (blue; *n* = 372, with 201 having PACS) and hospitalized patients (red; *n* = 17, with 11 having PACS). **k** Decision curve analysis of PACS score in derivation (left) and validation cohort (right) comparing the PACS score to a symptom-based score^14^ and clinical strategies of predicting none or all subjects with COVID-19 develop PACS. **l** Decision curve analysis of PACS score in hospitalized patients. **m** Estimated risk groups based on two probability thresholds (0.523 and 0.746) with corresponding positive (PPV) and negative (NPV) predictive values in the derivation cohort. Boxplots represent median (middle line) with upper and lower quartiles (box limits), and 1.5*interquartile ranges (whiskers). Variables were compared using a two-sided Wilcoxon’s test if not specified otherwise.

We extended the identified Ig signature to comprise additional parameters readily available during primary infection. Building on a previously published prediction model^14^, we considered patient age and number of symptoms during primary infection. For all continuous variables a linear relationship with the outcome PACS was accepted (Supplementary Fig. 2a). We found patient age and number of symptoms were increased in patients developing PACS (Fig. 3a and Supplementary Fig. 2b), whereas sex was not (Table 2). The symptom count during primary infection correlated with the maximal followed-up disease severity of COVID-19 patients (Fig. 3b). Vice versa, disease severity was associated with an increased risk of PACS (Supplementary Fig. 2c).

Regardless of their COVID-19 severity, 94% of individuals with a history of asthma bronchiale developed PACS and 71% developed post-COVID-19 syndrome defined as prolonged symptoms for more than 12 weeks after symptom onset. This is in stark contrast to 59% of individuals without a history of asthma bronchiale developing PACS and 42% developing post-COVID-19 syndrome (Fig. 3c). Interestingly, healthy controls and COVID-19 patients with a history of asthma bronchiale had lower serum IgG3 concentrations compared to their counterparts (Fig. 3d).

We applied our data obtained during primary infection to test different models predicting PACS. Use of a symptom-based score^14^, reliant on age, sex, and a number of symptoms during primary infection revealed an area under the curve (AUC) value of the receiver operating characteristic curve of 68% (CI 59–78%) and moderately underestimated the risk for PACS with a calibration-in-the-large of 1.76, a calibration slope of 0.57 and a Brier score of 0.328 (Fig. 3e). Based on our findings, we assessed previously identified predictors, such as patient age, sex, number of symptoms, body-mass-index, comorbidities, disease severity, and level of care as well as different combinations of serum Ig concentrations during primary infection to support development of a model predicting PACS (Supplementary Table 2). By combining patient age, number of symptoms during primary infection, history of asthma bronchiale, and an Ig signature consisting of IgM and IgG3 during primary infection, we were able to calculate a risk score—which we termed PACS score—that resulted in an AUC value of 77% (CI 69–85%) with a calibration-in-the-large of 0, a calibration slope of 1 and a Brier score of 0.185. To minimize overfitting, we modified the PACS score by shrinkage of the estimated coefficients. In a sensitivity analysis, the PACS score demonstrated, using the corresponding 6-month follow-up Ig measurements of our COVID-19 patients, a preserved calibration and ability to identify individuals with PACS with an AUC of 74% (CI 65–84%), a calibration-in-the-large of 0, a calibration slope of 1.2 and a Brier score of 0.191 (Fig. 3f). The addition of an interaction term between IgM and IgG3 significantly improved our PACS score (ANOVA; *p* = 0.02) compared to a model without interaction of IgM and IgG3 (Fig. 3g and Supplementary Tables 2 and 3).

Comparison of our PACS score to a recently published symptom-based score by Sudre et al.^14^ showed optimal performance of our PACS score in hospitalized patients of our cohort (Fig. 3h, i). We used our PACS score in an independent validation cohort of 395 individuals with confirmed COVID-19, including a small subgroup of hospitalized COVID-19 cases. This validated the improved predictive performance of our PACS score in the subgroup of hospitalized patients, resulting in an AUC of 99%, while the use of our PACS scores in the entire validation cohort resulted in an AUC of 64% (CI 58–69%) with a calibration-in-the-large of –0.3, a calibration slope of 0.8, and a Brier score of 0.239 (Fig. 3j and Supplementary Table 2). The PACS score performed well in the validation cohort, which consisted mainly of outpatients that showed a tendency to low IgG3 in individuals that had not recovered after 6 months (Supplementary Fig. 3a, b). Consistent with optimal performance in hospitalized patients, when applied to mild and severe COVID-19 patients, the PACS score performed better in the latter across all grades of severe COVID-19 (Supplementary Fig. 4a). Moreover, sensitivity analysis using different definitions of PACS showed a maintained ability of the PACS score to identify patients developing post-COVID-19 syndrome with symptoms lasting for more than 12 weeks and patients of the validation cohort that had not recovered after 6 months (Supplementary Fig. 4b).

Finally, we performed decision curve analyses, thus weighing the relative harms of false-positive and false-negative predictions. These decision curve analyses assessed the clinical utility of our PACS score and identified a range of threshold probabilities, in which the model could support clinical decision making compared to alternative intervention strategies, e.g. treating nobody or treating everybody with COVID-19^16^. The PACS score showed the best clinical utility within threshold probability ranges of 40% and 100% and an independently validated utility in ranges between 40% and 60% (Fig. 3k). Subgroup analysis in hospitalized patients revealed best clinical utility within probability threshold ranges of 35–100% and 55–100% in the derivation and validation cohort, respectively (Fig. 3l). Next, we calculated two probability thresholds as rule-in cut-offs for different clinical settings with the disparate prevalence of PACS. One threshold (0.52) was selected as optimal cut-off maximizing both sensitivity and specificity in the validation cohort. A second threshold (0.75) was calculated as the optimal cut-off for hospitalized patients of both derivation or validation cohorts independently (Supplementary Table 4). With a positive predictive value (PPV) of 0.88 in the derivation cohort and 0.90 in hospitalized patients, the upper threshold of 0.75 identifies with high specificity patients at very high risk for developing PACS. Conversely, with a PPV of 0.76 in the derivation cohort and 0.67 in outpatients, the lower threshold differentiates between patients at moderate versus high risk for developing PACS, while maintaining high sensitivity and negative predictive value (NPV) (Fig. 3m and Supplementary Table 4).

## Discussion

Collectively, we demonstrate that the development of PACS correlates with a distinct Ig signature as well as patient age, history of asthma bronchiale, and a number of symptoms, all measured during primary infection. We translated these findings into a model, termed PACS score. When applied to our cohort comprising 134 followed-up and extensively characterized COVID-19 patients, the PACS score performed better than a symptom-based score^14^, was independent of timepoint of testing and sex, and only required broadly available Ig measurements rather than specialized tests, such as SARS-CoV-2-specific immunoassays. Despite previous reports on female sex as a risk factor for PACS, male sex is associated with a worse outcome of acute COVID-19, and a sex-independent prediction score benefits from improved applicability to different healthcare settings^1,14^.

Compared to symptom-based prediction scores, the measurement of an Ig signature allows the identification of patients at risk for developing PACS, particularly, in hospitalized patients. This finding suggests a possible pathomechanism distinct from merely increased inflammation and immune activation. Moreover, unspecific Ig levels are stable over time, unlike inflammatory markers that only transiently increase early in the disease course. This biological stability of Igs further increases their utility as biomarkers, as independence of sampling timepoint facilitates clinical application and Ig signatures can be determined already before infection.

Limitations of our study comprise a non-excludable selection bias of patients enrolled in our study affecting the transferability of our findings to all SARS-CoV-2 RT-qPCR-positive patients, a non-excludable selection bias of patients agreeing to follow-up despite a high follow-up rate of 77%, as well as a limited number of hospitalized patients and differences in study design of the validation cohort. Moreover, our study included only a small number of participants of non-white ethnicity due to Central European demographics, potentially affecting the transferability of our findings.

Based on decision curve analyses we determined the highest clinical benefit of our PACS score to lie between threshold probability ranges of 40–60%, and above 55% in hospitalized patients, meaning that a clinician would advise preventive measures against PACS if the probability of PACS were above 55%. Thus, depending on future intervention strategies, associated side effects, and costs, our PACS score can be applied in a setting where false-positive predictions are of greater harm than false negatives. This would enable clinical studies and prevention strategies targeting high specificity patients at very high risk for developing PACS. We, therefore, suggest our PACS score can be applied to identify outpatients at risk, high-risk asthmatic patients, and hospitalized patients, the latter of which are already at high risk for developing PACS. Reliable identification of high-risk patients not only allows precise recommendation of early medical consultations but also facilitates the study of preventive treatment strategies, such as the use of inhaled corticosteroids in asthmatic and non-asthmatic patients and possibly intravenous Ig therapies^17,18^. Early measurement of Ig titers upon hospitalization of COVID-19 patients can support clinical decision-making and personalized treatment strategies.

In reflecting on the association of the identified Ig signature correlating with increased risk of PACS, the following aspects are worth considering. IgM and, particularly, IgG3 secretion by B cells is induced by interferons and antagonized by IL-4 signals^19–21^. Thus, reduced production of type I interferons, as proposed to occur in poorly controlled SARS-CoV-2 infection^22,23^, or a predisposition to secreting increased IL-4 concentrations, as present in asthma bronchiale^24^, may contribute to a failure to efficiently induce Ig isotype switching to IgG3. This hypothesis is consistent with our finding of low IgG3 in asthma bronchiale patients. Conversely, immune responses dominated by IgG3 can occur with similar temporal dynamics as IgM responses and have been associated with viral infections at mucosal tissues^25,26^. Thus, the reduced IgG3 concentrations in patients with PACS might support a role for IgG3 in Fc receptor-dependent viral control. Low IgG3 levels have also been linked to chronic fatigue syndrome, a debilitating condition resembling certain symptoms of PACS, as well as an increased rate of respiratory infections^18,27^.

PACS has been proposed to result from tissue damage due to direct effects of the virus, excessive inflammation, or thrombotic events; alternatively, PACS could be the consequence of bystander or virus-mediated activation of autoreactive T and B cells^28^. Recent observations of PACS resolution after SARS-CoV-2 vaccination might hint at the depletion of persisting viral reservoirs^29^. Our results highlight the benefit of measuring Igs for the early identification of patients at high risk for PACS, which in turn is crucial for understanding the pathomechanisms of PACS and identification of preventive measures for treatment and care.

## Methods

### Experimental study design

Adult individuals were included in the study and visited between April 2020 and August 2021. The study was approved by the Cantonal Ethics Committee of Zurich (BASEC #2016-01440). The majority of participants were of white ethnicity.

#### Coronavirus disease 2019 (COVID-19) patients

Following written informed consent, 175 patients with quantitative reverse-transcriptase quantitative polymerase chain reaction (RT-qPCR)-confirmed severe acute respiratory syndrome coronavirus 2 (SARS-CoV-2) infection were recruited for clinical evaluation and sampling of blood. Patients were included based on the selection criteria of SARS-CoV-2 PCR positivity and experiencing acute COVID-19. The multicentric study design comprised patient recruitment at four different hospitals in the area of Zurich, Switzerland, including the University Hospital Zurich (*n* = 111), the City Hospital Triemli Zurich (*n* = 35), the Limmattal Hospital (*n* = 15), and the Uster Hospital (*n* = 14). No exclusion criteria were applied on the analysis of the 175 COVID-19 patients, with the aim of generating a broadly applicable prediction score. Thus, SARS-CoV-2-specific RT-qPCR-positive individuals were included independently of comorbidities and medication. COVID-19 patients were sampled a first time during their primary SARS-CoV-2 infection (termed “primary infection”), a second time 6 months, and a third time one year after the initial blood sampling (Fig. 1). 39 patients declined follow-up or were not reachable and two patients were deceased. Eight patients only declined laboratory testing at 6-month follow-up and 12 patients at 1-year follow-up, whereas their medical history could be obtained by phone. In all followed-up COVID-19 patients (*n* = 134) medical history was obtained at least 3.5 months (105 days) after symptom onset to detect the presence or absence of PACS. Blood samples of COVID-19 patients were obtained during primary infection, around six months after symptom onset (*n* = 115) at an average of 199 days after symptom onset (interquartile range 185–216) and around one year after symptom onset (*n* = 38) at an average time point of 383 days (interquartile range 371–397) after symptom onset (Supplementary Fig. 5). The follow-up rate was 77% and followed-up patients are considered representative of the larger population of patients initially enrolled in the study (Supplementary Table 5).

#### Definitions

COVID-19 patients were grouped according to the World Health Organization classification criteria into (a) mild cases, comprising asymptomatic and symptomatic cases of mild illness and mild pneumonia, versus (b) severe cases, including severe pneumonia and acute respiratory distress syndrome (ARDS). Mild illness was defined as patients with uncomplicated respiratory tract infection and/or non-specific symptoms. Pneumonia was defined as the presence of respiratory symptoms, abnormal vital signs such as fever, and pathological lung examination findings, whereas patients with mild pneumonia showed no signs of severe pneumonia and did not require supplemental oxygen therapy. Severe pneumonia was defined as respiratory infection or fever with an observed respiratory rate greater than 30 breaths per minute, severe respiratory distress, and/or a SpO2 ≤ 93% on room air. Patients with severe pneumonia mostly required supplemental oxygen therapy. ARDS classification relied on measured oxygenation impairments (PaO2/FiO2a in mild ARDS ≤ 300 mmHg, moderate ARDS ≤ 200 mmHg, and severe ARDS ≤ 100 mmHg)^15,30,31^. Our COVID-19 derivation cohort did not contain any patients with sepsis or septic shock. For the validation cohort, patients with severe COVID-19 were identified as hospitalized patients reporting supplemental oxygen therapy during hospitalization. If not otherwise specified, all analyses were performed using the maximal followed-up disease severity of COVID-19 patients. We defined patients with PACS as individuals with PCR-confirmed COVID-19 experiencing one or more symptoms associated with COVID-19 that lasted for more than 4 weeks (i.e. 29 days and more) after the onset of the first COVID-19-related symptom^12^. Symptoms were assessed in a standardized manner by trained study physicians, both during primary infection (acute COVID-19) and at follow-up visits. During primary infection, five symptoms (fever, fatigue, cough, dyspnea, and gastrointestinal symptoms) were recorded systematically, which were subsequently used for our PACS prediction model. All five symptoms were patient-reported symptoms and, based on a standardized questionnaire, individually asked by a trained physician whether they were present during primary infection. Patient-reported temperature can be inaccurate for various reasons, including individual body temperature norms that vary with patient age as well as method and timepoint of measurement. Therefore, the following was considered as patient-reported “fever”: (i) reported increase of body temperature, (ii) fever chills, or (iii) sweating^32,33^. Gastrointestinal symptoms were counted as one symptom, also when multiple gastrointestinal symptoms were reported, including nausea, loss of appetite, heartburn, abdominal pain, flatulence, diarrhea, and obstipation. The severity of symptoms was not assessed. During follow-up visits, patients were asked whether and when they recovered from COVID-19 and which symptoms persisted. A total of nine symptoms were recorded systematically at follow-up visits (fever, cough, dyspnea, fatigue, gastrointestinal symptoms, headache, chest pain, anxiety and/or depression, and disorders of smell and/or taste; Table 1). Additional patient-reported prolonged symptoms were also recorded. Symptom severity was not assessed. When using the more stringent definition of PACS as symptoms lasting for more than 12 weeks, termed post-COVID-19 syndrome^12^, we found the preserved performance of our PACS prediction model (Supplementary Fig. 4b). For our validation cohort, we used the same definition of PACS as for our derivation cohort, i.e. patient-reported COVID-19-related symptoms lasting longer than four weeks, and we performed a sensitivity analysis showing preserved model performance using a different outcome based on whether patients had recovered after six months (Supplementary Fig. 4b).

#### Healthy controls

Following written informed consent, we additionally included 40 healthy controls who had no history of SARS-CoV-2 infection-associated symptoms, such as fever, rhinorrhea, respiratory symptoms (e.g. dry cough or shortness of breath), change in smell or taste, nausea, vomiting, and diarrhea^1^ and had a negative SARS-CoV-2 spike S1 protein-specific immunoassay, whereby individuals with borderline and positive values were excluded. Moreover, our healthy controls had no acute or active illness prior to or at blood sampling and no history of autoimmune disorder. We obtained a medical history from all 40 healthy controls at their blood sampling and at least 6 months thereafter in 35 healthy controls. Five healthy controls got infected with SARS-CoV-2 during the follow-up period and were therefore excluded from clinical follow-up (Supplementary Table 1). Participants were not compensated.

#### Validation cohort

Prognostic models were validated in a separate cohort of 395 PCR-confirmed COVID-19 patients that were enrolled at diagnosis between 06 August 2020 and 19 January 2021 and prospectively followed-up for 6 months after infection^34^. All serum samples were obtained during primary infection and in 98% of patients at 2 weeks after the diagnosis of COVID-19 with a median sampling time point of 19 days (interquartile range 17–22 days) after the onset of the first COVID-19-related symptom (Table 1). Pre-existing comorbidities and COVID-19 symptoms were recorded at baseline using standardized, self-administered, electronic questionnaires. Details regarding relevant medical history were clarified via phone by trained study personnel. Patient-reported symptoms were reassessed 1, 3, and 6 months after diagnosis. After 6 months, patients were asked whether they had recovered from COVID-19 or not.

### Immunoassays

All laboratory tests were performed in accredited laboratories at the University Hospital Zurich. Blood samples were collected in BD Vacutainer CAT serum tubes (Becton Dickinson; Cat# 367896). Different serum immunoglobulins subsets and IgG subclasses were measured using the commercially available turbidimetric Optilite^®^ assays using an Optilite^®^ analyzer (The Binding Site Group Ltd; Cat# NK004.OPT, NK006–NK010.OPT, NK012.OPT). Laboratory reference values are as follows (g/l): IgM (0.4–2.8), IgA (0.7–4.0), IgG (7.0–16.0), IgG1 (2.8–8.0), IgG2 (1.15–5.70), IgG3 (0.24–1.25), IgG4 (0.052–1.25). SARS-CoV-2-specific IgA and IgG antibodies were measured, as previously established^6^, by using a commercial enzyme-linked immunosorbent assay (ELISA) specific for the SARS-CoV-2 spike S1 protein (Euroimmun SARS-CoV-2 IgA and IgG immunoassay; Cat# EI 2606-9601A and G). Interleukin IL-6 and tumor necrosis factor (TNF) were quantified using R&D Systems assays (Cat# S6050 and LHSCM210, respectively). Antibody dilutions were prepared according to the manufacturer’s instructions. Blood samples obtained after SARS-CoV-2 vaccination were excluded from comparisons of SARS-CoV-2-specific Ig titers. We observed no sex differences in the measured total Igs and S1-specific antibody titers (Supplementary Fig. 6).

### Clinical prediction model

The sample for the development of our prediction model was obtained by including and following up all consecutive patients between April 2020 and August 2021 and resulted in a total of 134 followed-up patients. The number of outcome events was 85, which corresponds to the number of patients experiencing PACS. The required sample size for the development of clinical prediction models is a matter of active discussion and research. Our PACS score was developed using 14.2 events per predictor parameter, which is in line with the rule of thumb of 15 events per predictor parameter as well as several other recommendations on the required number of events per predictor parameter for accurate modeling in logistic regression analysis^35^. More precise estimates of the required sample size could be calculated based on published parameters of the previous studies^36^. However, as only one previous model for PACS prediction was available at the time of our study, and as definitions and prevalence of PACS in different populations vary significantly, we were unable to calculate precise requirements for model development. This might be reflected by some optimism in predictor effect estimates of our model (yielding a global shrinkage factor of 0.72) and a small overestimation of the overall risk for PACS (after shrinkage) in the validation cohort, that might be promoted by shrinkage of predictor effect estimates^37^.

Thus, the sample size was considered adequate to develop a prediction model with six predictor variables. These predictor variables were based on previous publications (age + number of symptoms during primary infection + history of asthma bronchiale)^14,38^ and include two new variables (total IgM + total IgG3) as well as one interaction term (total IgM * total IgG3), yielding 14.2 events per predictor parameter^35,39–43^. The validation cohort amounted to a sample size of 395 and counted 216 events, which was in line with a suggested sample size of 400 and an outcome event size of 200 in order to obtain precise calibration curves^44^.

The symptom-based prediction score was calculated using a previously published model^14^ and modified by applying it on five recorded symptoms instead of 14. The following five symptoms were recorded during primary infection: fever, fatigue, cough, shortness of breath (dyspnea), and gastrointestinal symptoms.

Our prediction model (PACS score) was built on a published prediction model^14^ that was based on “age + sex + number of symptoms during primary infection”. We have evaluated the three suggested predictors, together with other reported risk factors for PACS, such as asthma bronchiale^14,38^. Selection of new variables was a hypothesis-driven process based on the observation that total immunoglobulins are altered in COVID-19 patients experiencing long-term symptoms (Fig. 2), and previous studies connecting low total IgG3 levels to chronic fatigue syndrome and increased susceptibility to infection^18,45^. As some variables such as “age” represent risk factors for severe COVID-19 disease, a risk factor for PACS itself^38^, we further explored the influence of COVID-19 disease severity as well as associated risk factors (Table 2, Supplementary Table 2, and Supplementary Fig. 2c).

Moreover, we modified the estimated coefficients of the PACS score by shrinkage. As prognostic models tend to describe optimally the evaluated dataset but may perform less well in other datasets, we addressed this phenomenon of overfitting by applying the statistical method of shrinkage. Estimated coefficients of the generalized linear model were multiplied with a global shrinkage factor (0.72) that was calculated using the dfbeta-method^46,47^. Original and regression coefficients after shrinkage are summarized in Supplementary Table 3 with 95% confidence intervals (CI) and corresponding *p* values. Areas under the curve (AUC) of receiver operating characteristic (ROC) curves and calibration plots were calculated as previously described^48,49^. The PACS score was validated in a separate validation cohort using the same patient-centered outcome definition for PACS as in the derivation cohort. The PACS score (after shrinkage) can be calculated and the logistic regression model reproduced using the following R code: PACS_score < - glm(PACS_score ~ –1 + offset(–0.981011 + 0.2616998*scale(age)+0.3307986*number of symptoms during primary infection + 1.896502*history of asthma bronchiale + 0.8429766*total IgM (g/l) + 1.3716198*total IgG3 (g/l)–1.5316550*IgM*IgG3), family = binomial, data = patient_data_to_test), with the variables “age” in years, “number of symptoms during primary infection” ranging from zero to five, and “history of asthma bronchiale” as number zero if absent and number one if present. Individual risk for PACS can further be predicted using the following R code: predictions < −predict(PACS_score, patient_data_to_test, type = “response”). The number of symptoms can be determined by counting the occurrence of the following five symptom categories in tested COVID-19 patients (all self-reported): fever, fatigue, cough, shortness of breath (dyspnea), and gastrointestinal symptoms.

### Statistics

Descriptive statistics for the followed-up healthy controls, COVID-19 patients, and validation cohort are presented as numbers and percentages of the total for categorical variables, as well as the median and interquartile range (IQR) for continuous variables. Comparison of variables was performed using non-parametric Wilcoxon’s rank-sum test if not otherwise specified. Evidence was quantified on a continuous scale, as results were considered exploratory. Thus, p values are to be interpreted as quantified evidence of the hypothesis without specified significance thresholds. In Table 2, odds ratios of categorical variables were calculated by median-unbiased estimation and odds ratios of continuous variables were calculated using univariate, unadjusted regression models for the outcome PACS. Horizontal lines in split violin plots indicate median values. Wedge sizes of radar plots visualize median values of measured immunoglobulins in patients with or without PACS, normalized by dividing the respective median with the overall median measured in COVID-19 patients. Microsoft Office Excel (version 2102) was used for data collection. Statistical analyses were performed with R (version 4.1.2) and using the packages “biostatUZH” (version 1.8.0), “CalibrationCurves” (version 0.1.2), “dcurves” (version 0.2.0), “epitools” (version 0.5-10.1), “interactions” (version 1.1.5), “gbm” (version 2.1.8), “ggstatsplot” (version 0.9.0), “interactions”, “pROC” (version 1.18.0), “mgcv” (version 1.8-38), “shrink” (version 1.2.1), and “sjPlot” (version 2.8.9), and missing values were omitted. The present study is reported according to the STROBE (Statement for reporting cohort studies) and TRIPOD (Statement for reporting clinical prediction models) guidelines^50,51^.

### Reporting summary

Further information on research design is available in the Nature Research Reporting Summary linked to this article.

## Supplementary information

Supplementary Information

Description of Additional Supplementary Files

Supplementary Software 1

Reporting Summary

## Data Availability

All relevant data generated in this study are provided in the Supplementary Information. A PACS score calculator is accessible online (www.pacs-score.com). Source data are provided with this paper.

## Source data

Source Data

## Code availability

R code for immunoglobulin signature analysis and prediction model development is provided in the Supplementary Software 1.

## References

[1] Wiersinga WJ, Rhodes A, Cheng AC, Peacock SJ, Prescott HC (2020). Pathophysiology, transmission, diagnosis, and treatment of coronavirus disease 2019 (COVID-19): a review. JAMA.

[2] Silvin A (2020). Elevated calprotectin and abnormal myeloid cell subsets discriminate severe from mild COVID-19. Cell.

[3] Schulte-Schrepping J (2020). Severe COVID-19 is marked by a dysregulated myeloid cell compartment. Cell.

[4] Chevrier S (2021). A distinct innate immune signature marks progression from mild to severe COVID-19. Cell Rep. Med..

[5] To KK (2020). Temporal profiles of viral load in posterior oropharyngeal saliva samples and serum antibody responses during infection by SARS-CoV-2: an observational cohort study. Lancet Infect. Dis..

[6] Cervia C (2021). Systemic and mucosal antibody responses specific to SARS-CoV-2 during mild versus severe COVID-19. J. Allergy Clin. Immunol..

[7] Blanco-Melo D (2020). Imbalanced host response to SARS-CoV-2 drives development of COVID-19. Cell.

[8] van Kampen JJA (2021). Duration and key determinants of infectious virus shedding in hospitalized patients with coronavirus disease-2019 (COVID-19). Nat. Commun..

[9] Office for National Statistics (ONS). *Prevalence of Long COVID Symptoms and COVID-19 Complications* https://www.ons.gov.uk/peoplepopulationandcommunity/healthandsocialcare/healthandlifeexpectancies/datasets/prevalenceoflongcovidsymptomsandcovid19complications (2020).

[10] National Institute for Health Research (NIHR). *Living with Covid 19—Second Review* https://evidence.nihr.ac.uk/themedreview/living-with-covid19-second-review/, 10.3310/themedreview_45225 (2021).

[11] Menges, D. et al. Burden of post-COVID-19 syndrome and implications for healthcare service planning: a Population-based Cohort Study. *PLoS ONE*10.1371/journal.pone.0254523 (2021). 10.1371/journal.pone.0254523 PMC8274847 34252157

[12] Shah, W., Hillman, T., Playford, E. D. & Hishmeh, L. Managing the long term effects of covid-19: summary of NICE, SIGN, and RCGP rapid guideline. *BMJ* **372**, (2021) 10.1136/bmj.n136. 10.1136/bmj.n136 33483331

[13] Lambert, N. et al. COVID-19 survivors’ reports of the timing, duration, and health impacts of post-acute sequelae of SARS-CoV-2 (PASC) infection. Preprint at *medRxiv*10.1101/2021.03.22.21254026 (2021).

[14] Sudre, C. H. et al. Attributes and predictors of long COVID. *Nat. Med.*10.1038/s41591-021-01292-y (2021). 10.1038/s41591-021-01292-y PMC7611399 33692530

[15] WHO. COVID-19 Clinical management: living guidance. World Health Organization www.who.int/publications/i/item/WHO-2019-nCoV-clinical-2021-1 (2021).

[16] Vickers AJ, Elkin EB (2006). Decision curve analysis: a novel method for evaluating prediction models. Med. Decis. Mak..

[17] Ramakrishnan S (2021). Inhaled budesonide in the treatment of early COVID-19 (STOIC): a phase 2, open-label, randomised controlled trial. Lancet Respir. Med..

[18] Scheibenbogen C (2021). Tolerability and efficacy of s.c. IgG self-treatment in ME/CFS patients with IgG/IgG subclass deficiency: a Proof-of-Concept Study. J. Clin. Med..

[19] Snapper CM (1992). Induction of IgG3 secretion by interferon gamma: a model for T cell-independent class switching in response to T cell-independent type 2 antigens. J. Exp. Med..

[20] Le Bon A (2001). Type I interferons potently enhance humoral immunity and can promote isotype switching by stimulating dendritic cells in vivo. Immunity.

[21] Deenick EK, Hasbold J, Hodgkin PD (2005). Decision criteria for resolving isotype switching conflicts by B cells. Eur. J. Immunol..

[22] Hadjadj J (2020). Impaired type I interferon activity and inflammatory responses in severe COVID-19 patients. Science.

[23] Sprent J, King C (2021). COVID-19 vaccine side effects: the positives about feeling bad. Sci. Immunol..

[24] Akdis CA (2020). Type 2 immunity in the skin and lungs. Allergy.

[25] Hjelholt A, Christiansen G, Sørensen US, Birkelund S (2013). IgG subclass profiles in normal human sera of antibodies specific to five kinds of microbial antigens. Pathog. Dis..

[26] Lemos MP (2016). In men at risk of HIV infection, IgM, IgG1, IgG3, and IgA reach the human foreskin epidermis. Mucosal Immunol..

[27] Kedor, C. et al. Chronic COVID-19 Syndrome and Chronic Fatigue Syndrome (ME/CFS) following the first pandemic wave in Germany—a first analysis of a prospective observational study. Preprint at *medRxiv*10.1101/2021.02.06.21249256 (2021).

[28] Akbar, A. et al. Report: long-term immunological health consequences of COVID-19. *Br. Soc. Immunol.* www.immunology.org/sites/default/files/BSI_Briefing_Note_August_2020_FINAL.pdf (2020).

[29] Arnold, D. et al. Are vaccines safe in patients with Long COVID? A prospective observational study. *medRxiv* (2021), 10.1101/2021.03.11.21253225.30.

[30] WHO. *COVID-19 Clinical Management: Interim Guidance* (World Health Organization, 2021).

[31] ARDS-Definition-Task-Force. (2012). Acute respiratory distress syndrome: the Berlin Definition. JAMA.

[32] Shann F, Mackenzie A (1996). Comparison of rectal, axillary, and forehead temperatures. Arch. Pediatr. Adolesc. Med..

[33] Quer G (2021). Wearable sensor data and self-reported symptoms for COVID-19 detection. Nat. Med..

[34] ISRCTN registry. *Zurich Coronavirus Cohort: an Observational Study to Determine Long-term Clinical Outcomes and Immune Responses After Coronavirus Infection (COVID-19), Assess the Influence of Virus Genetics, and Examine the Spread of the Coronavirus in the Population of the Canton of Zurich, Switzerland*10.1186/ISRCTN14990068 (2020).

[35] Harrel, F. E. Regression modeling strategies. *hbiostat* https://hbiostat.org/doc/rms.pdf (2021).

[36] Riley RD (2019). Minimum sample size for developing a multivariable prediction model: PART II - binary and time-to-event outcomes. Stat. Med..

[37] Riley RD (2021). Penalization and shrinkage methods produced unreliable clinical prediction models especially when sample size was small. J. Clin. Epidemiol..

[38] Blomberg, B. et al. Long COVID in a prospective cohort of home-isolated patients. *Nat. Med*. 10.1038/s41591-021-01433-3 (2021). 10.1038/s41591-021-01433-3 PMC8440190 34163090

[39] Harrell FE, Lee KL, Califf RM, Pryor DB, Rosati RA (1984). Regression modelling strategies for improved prognostic prediction. Stat. Med..

[40] Harrell FE, Lee KL, Matchar DB, Reichert TA (1985). Regression models for prognostic prediction: advantages, problems, and suggested solutions. Cancer Treat. Rep..

[41] Peduzzi P, Concato J, Feinstein AR, Holford TR (1995). Importance of events per independent variable in proportional hazards regression analysis II. Accuracy and precision of regression estimates. J. Clin. Epidemiol..

[42] Peduzzi P, Concato J, Kemper E, Holford TR, Feinstein AR (1996). A simulation study of the number of events per variable in logistic regression analysis. J. Clin. Epidemiol..

[43] Vittinghoff E, McCulloch CE (2007). Relaxing the Rule of Ten events per variable in logistic and Cox regression. Am. J. Epidemiol..

[44] Van Calster B, McLernon DJ, van Smeden M, Wynants L, Steyerberg EW (2019). Calibration: the Achilles heel of predictive analytics. BMC Med..

[45] Löbel M (2015). Polymorphism in COMT is associated with IgG3 subclass level and susceptibility to infection in patients with chronic fatigue syndrome. J. Transl. Med..

[46] Dunkler D, Sauerbrei W, Heinze G (2016). Global, parameterwise and joint shrinkage factor estimation. J. Stat. Softw..

[47] Held U (2018). Prognostic function to estimate the probability of meaningful clinical improvement after surgery—results of a prospective multicenter observational cohort study on patients with lumbar spinal stenosis. PLoS ONE.

[48] Robin X (2011). pROC: an open-source package for R and S+ to analyze and compare ROC curves. BMC Bioinforma..

[49] Spiegelhalter DJ (1986). Probabilistic prediction in patient management and clinical trials. Stat. Med..

[50] Collins GS, Reitsma JB, Altman DG, Moons KGM (2015). Transparent reporting of a multivariable prediction model for individual prognosis or diagnosis (TRIPOD): the TRIPOD statement. BMJ.

[51] Von Elm E (2007). Strengthening the reporting of observational studies in epidemiology (STROBE) statement: guidelines for reporting observational studies. BMJ.

