# Supplementary material for: Immunoglobulin signature predicts risk of post-acute COVID-19 syndrome
Source: Nat Commun. 2022 Jan 25;13:446. doi: 10.1038/s41467-021-27797-1 (PMC8789854; doi:10.1038/s41467-021-27797-1)
Supplement: Supplementary file 1 — Supplementary Information [file 41467_2021_27797_MOESM1_ESM.pdf]

Supplementary material:

Immunoglobulin signature predicts risk of post-acute COVID-19 syndrome

Authors: Carlo Cervia[1], Yves Zurbuchen[1], Patrick Taeschler[1], Tala Ballouz[2], Dominik Menges[2], Sara Hasler[1], Sarah Adamo[1], Miro E. Raeber[1], Esther Bächli[3], Alain Rudiger[4], Melina Stüssi-Helbling[5], Lars C. Huber[5], Jakob Nilsson[1], Ulrike Held[2], Milo A. Puhan[2], and Onur Boyman[1,6]*

Affiliations:

[1] Department of Immunology, University Hospital Zurich, University of Zurich; Zurich, Switzerland.

[2] Epidemiology, Biostatistics and Prevention Institute, University of Zurich; Zurich, Switzerland.

[3] Clinic for Internal Medicine, Uster Hospital; Uster, Switzerland.

[4] Department of Medicine, Limmattal Hospital; Schlieren, Switzerland.

[5] Clinic for Internal Medicine, City Hospital Triemli Zurich; Zurich, Switzerland.

[6] Faculty of Medicine, University of Zurich; Zurich, Switzerland.

* Corresponding author. Email: onur.boyman@uzh.ch.

Supplementary Table 1. History of respiratory disease and SARS-CoV-2 infection during follow-up in study participants (*n* = 174).

Disease severity	**Control group (*n* = 40)**	**Mild COVID-19 (*n* = 89)**	**Severe COVID-19 (*n* = 45)**
Respiratory symptoms – no. (%)	13 (32.5)	26 (29.2)	7 (15.6)
SARS-CoV-2 exposure[a] – no. (%)	8 (20.0)	14 (15.7)	4 (8.9)
Respiratory symptoms upon SARS-CoV-2 exposure – no. (%)	2 (5.0)	0 (0)	0 (0)
Positive SARS-CoV-2-specific RT-qPCR test – no. (%)	5 (12.5)	0 (0)	0 (0)
Negative SARS-CoV-2-specific test[b] – no. (%)	13 (32.5)	31 (34.8)	13 (28.9)

[a] Exposure to SARS-CoV-2-positive individual without adequate safety measures
[b] SARS-CoV-2-specific RT-qPCR or antigen rapid testing
Abbreviations: RT-qPCR, reverse-transcriptase quantitative polymerase chain reaction.

Supplementary Table 2. Logistic regression models for prediction of PACS. Different logistic regression models were applied on all patients of the derivation (*n* = 134) and validation cohort (*n* = 389), outpatients of the derivation (*n* = 80) and validation cohort (*n* = 372), and hospitalized patients of the derivation (*n* = 54) and validation cohort (*n* = 17). Area under the curve (AUC) values and 95% confidence intervals (CI) of the receiver operating characteristic curve are listed for the prediction of PACS. The color scheme reflects the performance of different scores and shows the lowest (light green) and highest (dark green) AUC in each subgroup analysis of the patient cohorts (columns).

Predictor variables	Derivation cohort			Validation cohort		
Prediction model - AUC (CI)	**All**	**Out-patients**	**Hospitalized**	**All**	**Out-patients**	**Hospitalized**
Age + Sex + No. of symptoms[a,14]	683 (586-780)	614 (488-739)	569 (341-798)	587 (530-644)	582 (523-640)	712 (446-979)
Age	643 (543-745)	568 (439-697)	460 (219-700)	513 (454-571)	511 (451-570)	591 (270-912)
Age + Sex	648 (547-748)	561 (433-689)	544 (304-785)	535 (477-593)	533 (474-592)	530 (221-839)
Age + No. of symptoms	705 (610-799)	690 (573-806)	595 (385-804)	629 (574-685)	621 (564-679)	848 (580-1000)
Age + No. of symptoms + Lung disease[b]	748 (660-835)	740 (633-846)	632 (428-836)	635 (579-690)	627 (570-684)	848 (580-1000)
Age + No. of symptoms + Asthma bronchiale[c]	754 (668-839)	735 (627-842)	648 (445-851)	643 (589-698)	636 (580-692)	833 (564-1000)
Age + Disease severity (at sampling timepoint)[d]	656 (559-754)	568 (439-697)	568 (358-779)			
Age + Disease severity (followed-up)	668 (572-764)	574 (446-702)	602 (385-820)	521 (463-580)	511 (451-570)	583 (269-897)
Age + Disease severity (followed-up) + Asthma bronchiale	727 (640-815)	655 (535-774)	671 (476-865)	553 (495-610)	544 (485-603)	583 (269-897)
Age + Disease grades[e] (followed-up) + Asthma bronchiale	749 (666-832)	675 (558-792)	753 (586-921)			
Age + No. of symptoms + Asthma bronchiale + Body-Mass-Index	739 (644-834)	738 (622-855)	647 (444-850)	643 (589-698)	631 (574-688)	833 (564-1000)
Age + No. of symptoms + Asthma bronchiale + IgM[f]	751 (666-837)	726 (617-835)	647 (450-845)	639 (584-694)	631 (574-687)	894 (725-1000)
Age + No. of symptoms + Asthma bronchiale + IgA	753 (668-838)	733 (624-842)	662 (469-856)	641 (587-696)	634 (578-691)	833 (598-1000)
Age + No. of symptoms + Asthma bronchiale + IgG1	754 (669-840)	734 (626-842)	649 (445-854)	643 (588-698)	636 (579-692)	833 (564-1000)
Age + No. of symptoms + Asthma bronchiale + IgG2	756 (670-842)	743 (636-850)	649 (442-856)	644 (589-699)	638 (581-694)	848 (606-1000)
Age + No. of symptoms + Asthma bronchiale + IgG3	756 (671-842)	745 (639-851)	647 (452-843)	642 (587-697)	635 (579-691)	879 (687-1000)
Age + No. of symptoms + Asthma bronchiale + IgG4	756 (670-842)	737 (629-845)	651 (445-857)	643 (588-698)	636 (580-692)	848 (580-1000)
Age + No. of symptoms + Asthma bronchiale + IgM + IgG3	753 (668-839)	737 (629-846)	651 (461-841)	636 (581-691)	628 (572-685)	879 (705-1000)
Age + No. of symptoms + Asthma bronchiale + IgM + IgG2 + IgG3	754 (668-839)	737 (629-846)	647 (456-838)	637 (582-692)	629 (573-686)	894 (726-1000)
Age + No. of symptoms + Asthma bronchiale + IgM * IgG2	754 (669-839)	741 (634-849)	660 (465-856)	625 (570-681)	617 (560-674)	879 (713-1000)
**Age + No. of symptoms + Asthma bronchiale + IgM * IgG3**	771 (691-851)	723 (613-834)	741 (580-902)	636 (581-691)	626 (569-683)	985 (943-1000)
Age + Fatigue[g] + Asthma bronchiale + IgM * IgG2	745 (661-829)	673 (556-790)	735 (569-902)	603 (547-660)	593 (535-651)	764 (456-1000)
Age + No. of symptoms + Level of care (at sampling timepoint)	754 (668-839)	735 (627-842)	648 (445-851)	643 (589-698)	636 (580-692)	833 (564-1000)
Age + No. of symptoms + Level of care (at sampling timepoint) + IgM	751 (665-836)	725 (615-834)	645 (446-845)	639 (584-694)	630 (574-687)	894 (725-1000)
Age + No. of symptoms + Level of care (at sampling timepoint) + IgG3	756 (670-841)	744 (638-851)	647 (452-843)	642 (587-696)	635 (578-691)	879 (687-1000)

[a] No. of symptoms during primary infection (0-5; fever, fatigue, cough, dyspnea, and gastrointestinal symptoms)
[b] History of lung disease
[c] History of asthma bronchiale
[d] Disease severity (mild or severe COVID-19)
[e] Disease grades (asymptomatic, mild illness, mild pneumonia, severe pneumonia, mild ARDS, moderate ARDS, severe ARDS)
[f] Total Ig titers [g/l]
[g] Presence of fatigue during primary infection (yes or no)
PACS score is shown in bold.

Supplementary Table 3. Variables of the PACS score. Estimated coefficients and their 95% confidence intervals, p values, and coefficients after shrinkage are listed for the prediction of PACS (logistic regression model), including an interaction term between total IgM and IgG3 titers (IgM * IgG3). The global shrinkage factor was 0.72.

Variables	Coefficients = log odds ratios	95% confidence interval	p value	Coefficients after shrinkage
Intercept	-1.532	-3.74 to 0.36	0.139	-0.981
Patient age [years]	0.364	-0.04 to 0.79	0.085	0.262
No. of symptoms during primary SARS-CoV-2 infection [0-5]	0.460	0.11 to 0.84	0.014	0.331
History of asthma bronchiale [no/yes]	2.634	0.83 to 5.64	0.019	1.897
Total IgM (g/l)	1.171	-0.16 to 2.82	0.121	0.843
Total IgG3 (g/l)	1.905	-0.19 to 4.58	0.116	1.372
IgM * IgG3	-2.128	-4.45 to -0.29	0.044	-1.532

Supplementary Table 4. Model behavior at different probability thresholds. Two thresholds were selected for further examination. 0.523 was selected as optimal cut-off maximizing both sensitivity and specificity in the validation cohort. 0.746 was calculated as optimal cut-off maximizing both sensitivity and specificity in hospitalized patients of the derivation cohort (*n* = 134, with 85 having PACS) and hospitalized patients of the validation cohort (*n* = 389, with 212 having PACS).

Cohort	Sensitivity	Specificity	PPV	NPV
**Rule-in cut-off for general population and outpatients (0.523)**				
Derivation cohort	0.835	0.551	0.763	0.659
- Outpatients	0.705	0.583	0.674	0.618
Validation cohort	0.797	0.395	0.612	0.619
- Outpatients	0.786	0.409	0.610	0.619
**Rule-in cut-off for general population and hospitalized patients (0.746)**				
Derivation cohort	0.518	0.878	0.878	0.512
- Hospitalized patients	0.634	0.769	0.897	0.400
Validation cohort	0.283	0.870	0.723	0.503
- Hospitalized patients	0.909	1.000	1.000	0.857

Supplementary Table 5. Participant characteristics of entire derivation cohort, including followed-up and non-followed-up patients.

Demographics	Whole cohort (*n* = 175)	Followed-up patients (*n* = 134)	Non-followed-up patients (*n* = 41)
Severe COVID-19 cases – no. (%)	65 (37.1)	45 (33.6)	20 (48.8)
Age – median (IQR) [yrs]	47 (31–67)	43 (30–64)	66 (34–78)
Sex – male no. (%)	94 (53.7)	75 (56.0)	19 (46.3)
Level of care[a] – hospitalized no. (%)	81 (46.3)	54 (40.3)	27 (65.9)
No. of symptoms[b] (IQR)	2 (1–3)	2 (1–3)	2 (2–3)
**Pre-existing comorbidities and treatments** – no. (%)			
Cardiovascular disease	32 (18.3)	18 (13.4)	14 (34.1)
Diabetes mellitus	25 (14.3)	19 (14.2)	6 (14.6)
Hypertension	50 (28.6)	31 (23.1)	19 (46.3)
Kidney disease	25 (14.3)	15 (11.2)	10 (24.4)
Lung disease	26 (14.9)	21 (15.7)	5 (12.2)
Asthma bronchiale	17 (9.7)	17 (12.7)	0
Malignancy	12 (6.9)	8 (6.0)	4 (9.8)
Systemic immunosuppression	10 (5.7)	9 (6.7)	1 (2.4)
**Laboratory characteristics at primary infection**			
Time after symptom onset [days]	11 (7–16)	11 (7–17)	13 (7–15)
CRP [mg/L]	7.2 (0.9–55.5)	4.4 (0.7–45.0)	25.3 (1.6–78.3)
IL-6 [pg/mL]	3.9 (0.8–18.6)	3.6 (0.4–16.3)	6.9 (1.6–30.7)
TNF [pg/mL]	11.1 (8.6–16.2)	11.0 (8.6–15.5)	14.5 (8.3–19.1)
Leukocytes [10^9/L]	5.6 (4.5–7.0)	5.9 (4.6–7.1)	5.2 (3.8–6.3)
Neutrophils [10^9/L]	3.4 (2.4–4.5)	3.4 (2.6–4.5)	2.7 (2.2–4.4)
Lymphocytes [10^9/L]	1.4 (0.9–2.0)	1.6 (1.0–2.1)	1.0 (0.7–1.6)
NLR	2.0 (1.4–3.8)	2.0 (1.4–3.8)	2.2 (1.2–3.9)
SARS-CoV-2 IgA [OD ratio]	3.2 (0.8–7.4)	3.0 (0.8–7.3)	3.7 (0.8–7.9)
SARS-CoV-2 IgG [OD ratio]	1.1 (0.3–5.1)	1.0 (0.3–4.6)	1.2 (0.3–6.6)
Total IgM [g/l]	1.0 (0.7–1.4)	1.0 (0.7–1.4)	1.1 (0.7–1.3)
Total IgA [g/l]	2.3 (1.7–3.0)	2.3 (1.7–3.0)	2.4 (1.6–2.9)
Total IgG [g/l]	11.0 (9.3–12.9)	11.2 (9.4–13.0)	10.7 (7.9–12.0)
Total IgG1 [g/l]	5.7 (4.4–6.9)	5.9 (4.4–6.9)	5.5 (4.4–6.9)
Total IgG2 [g/l]	3.7 (2.6–4.6)	3.7 (2.9–4.7)	3.2 (2.2–4.6)
Total IgG3 [g/l]	0.7 (0.5–1.0)	0.7 (0.5–1.1)	0.7 (0.5–1.0)
Total IgG4 [g/l]	0.3 (0.2–0.5)	0.3 (0.2–0.5)	0.3 (0.2–0.4)

No information on BMI of non-followed-up patients available.
[a] Level of care at sampling timepoint
[b] No. of symptoms during primary infection (0-5; fever, fatigue, cough, dyspnea, and gastrointestinal symptoms)

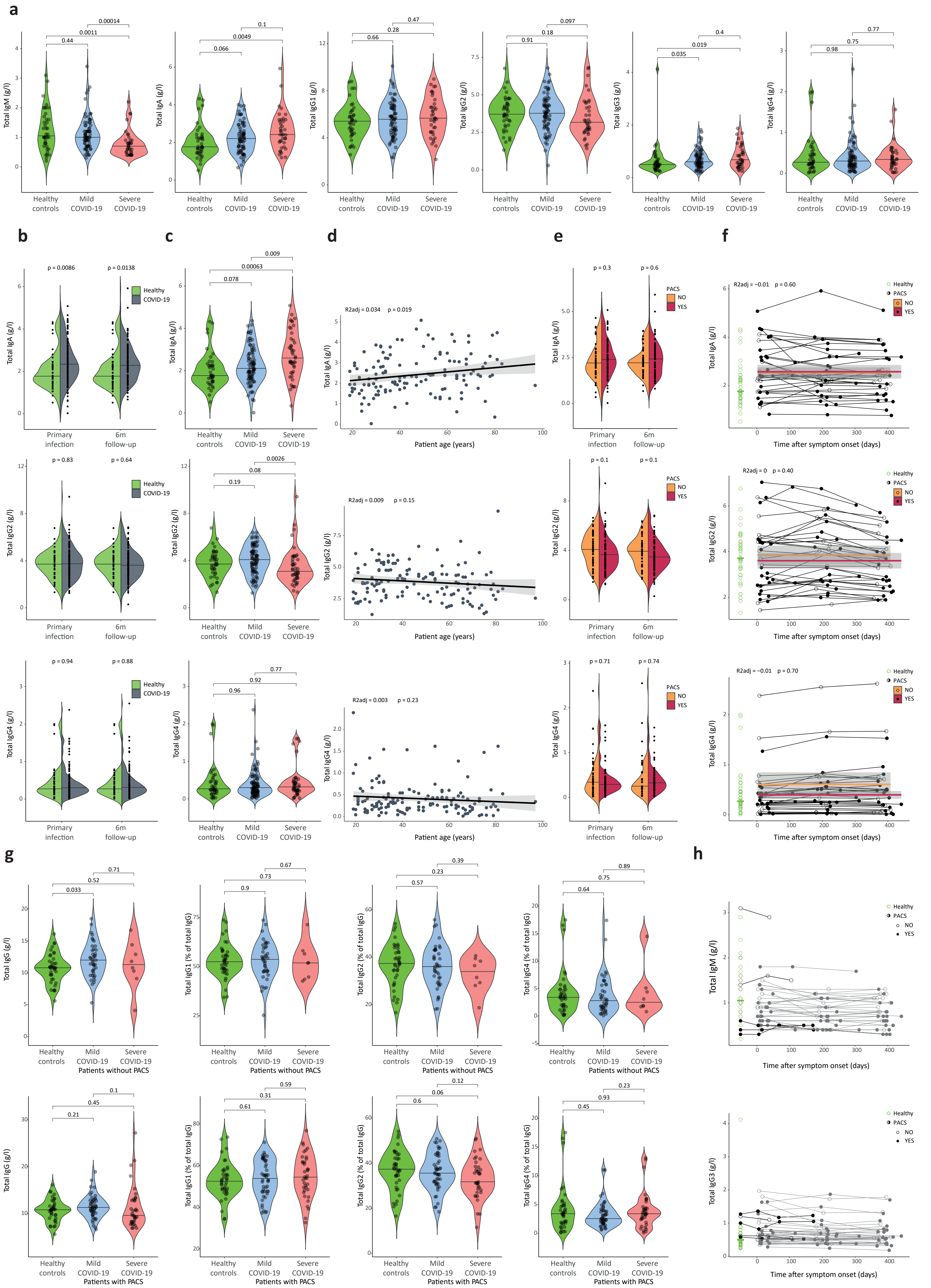

Supplementary Figure 1

Supplementary Fig. 1. Immunoglobulin signatures in COVID-19 patients. a, Total serum concentrations of IgM, IgA, and IgG subclasses IgG1 to IgG4 in healthy controls (*n* = 40) versus COVID-19 patients with mild (*n* = 77) or severe disease (*n* = 38) at 6-month (6m) follow-up. **b-c**, Total serum concentrations of IgA, IgG2, and IgG4 in healthy controls versus (b) all (*n* = 134) or (c) mild (*n* = 89) and severe (*n* = 45) COVID-19 cases during primary infection. **d**, Ig titers at primary infection as a function of age in COVID-19 patients, with adjusted R2 (R2adj) and p values of linear model (shown with 95% confidence interval [CI]). **e**, Ig signatures in patients without or with PACS, during primary infection (*n* = 49 and 85, respectively) and 6-month follow-up (*n* = 41 and 74, respectively). **f**, Ig titers in patients attending all follow-up visits (*n* = 34) as a function of days after symptom onset, with R2adj and p values of generalized additive model (shown with 95% CI). Corresponding patients without (circles; *n* = 12) and with PACS (dots; *n* = 22) are connected, with a spline overlaid for both groups (orange and red, respectively). Green horizontal line indicates median in healthy controls (left). **g**, Total serum concentration of IgG, and percentages of IgG1, IgG2, and IgG4 of total IgG during primary infection in healthy controls, mild and severe COVID-19 patients without (top) or with PACS (bottom). **h**, IgM (top) and IgG3 (bottom) titers in patients attending all follow-up visits (*n* = 34) as a function of days after symptom onset, with additional 5 patients that were initially included as healthy controls and later as COVID-19 patients upon SARS-CoV-2 infection. Individuals not developing and developing PACS are visualized as circles or dots respectively. Variables were compared using two-sided Wilcoxon's test.

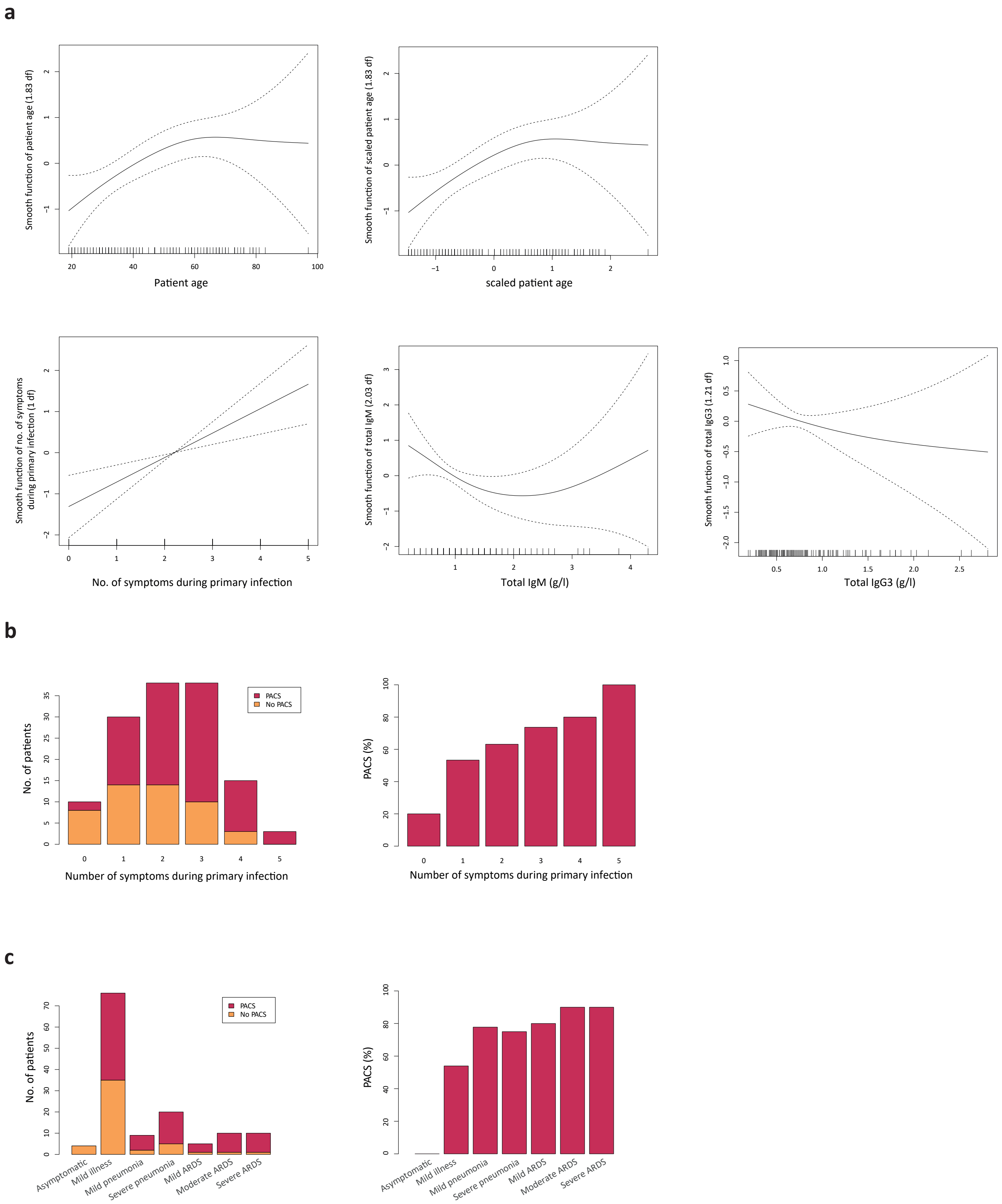

Supplementary Figure 2

Supplementary Fig. 2. Predictor variables of PACS. **a**, Linearity of continuous variables included in PACS score: age, age translated into terms of a scale of age norms, number of symptoms during primary infection (0-5; fever, fatigue, cough, dyspnea, and gastrointestinal symptoms), total IgM (g/l), and total IgG3 (g/l) during primary infection. Variables were examined as univariate functions of the binomial outcome PACS using a generalized additive model (n = 134, with 85 having PACS). **b**, Proportion (left) and percentages (right) of patients with PACS in patients reporting zero to five symptoms during primary infection. **c**, Proportion (left) and percentages (right) of patients with PACS in patients with different followed-up disease grades.

a

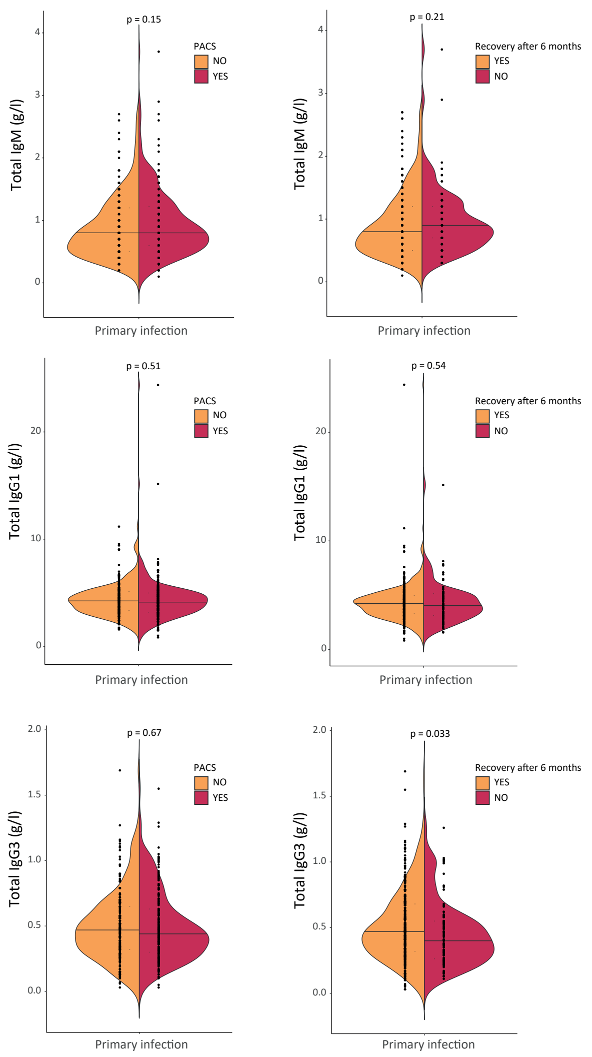

b

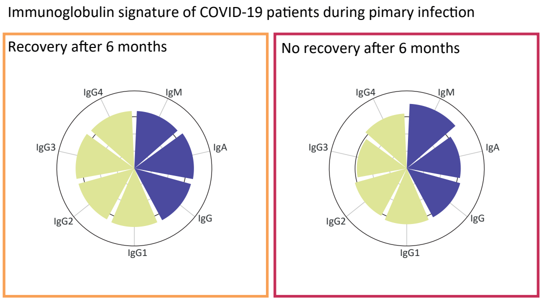

Supplementary Figure 3

Supplementary Fig. 3. Total immunoglobulin titers during primary infection in validation cohort. **a**, Total IgM, IgG1 and IgG3 titers in patients without or with PACS, (*n* = 177 and 212, respectively) as well as patients that reported full recovery or no full recovery after six months (right; *n* = 299 and 90, respectively). Variables were compared using two-sided Wilcoxon's test. **b**, Radar plots with wedge sizes representing median Ig concentrations of patients without or with recovery after six months, normalized to median concentrations of all patients.

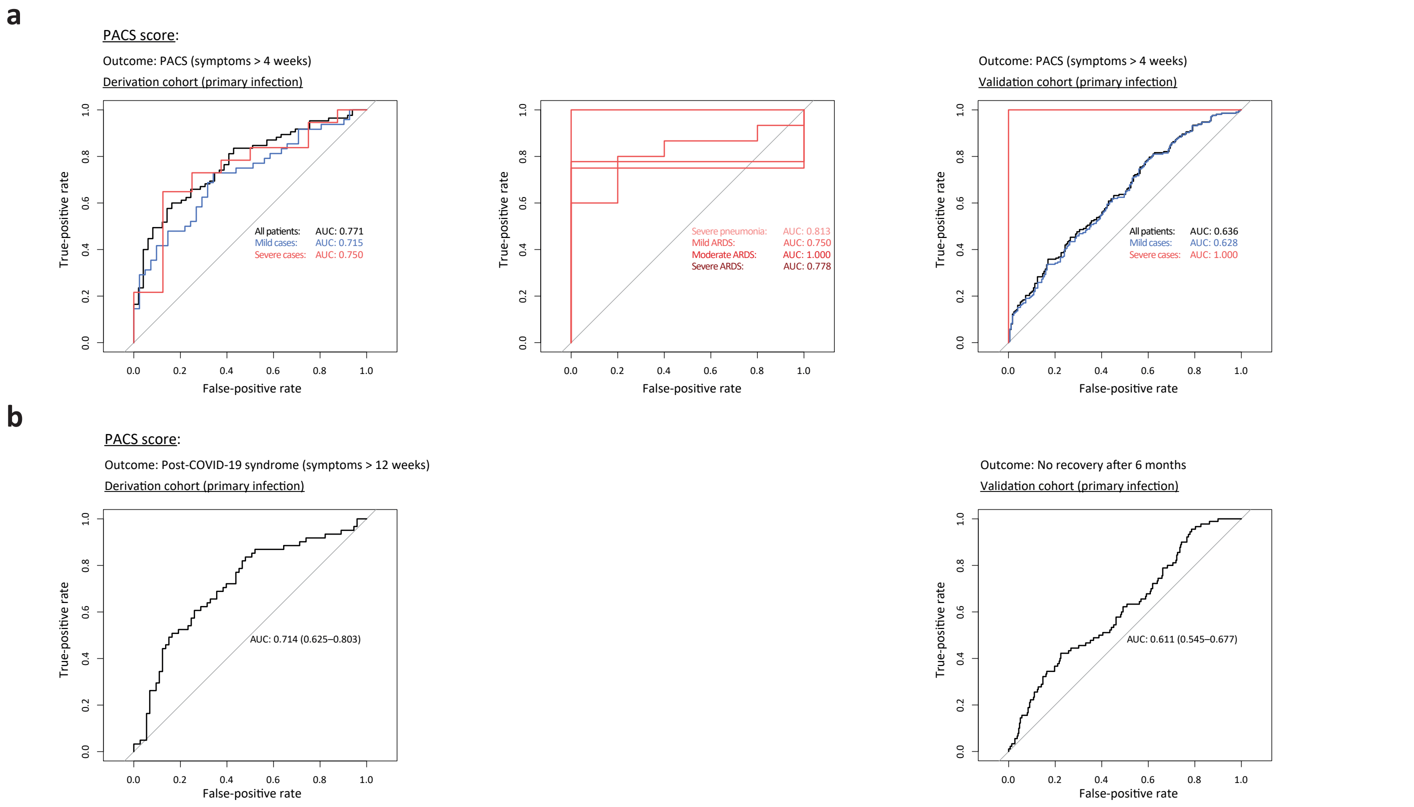

Supplementary Figure 4

Supplementary Fig. 4. PACS score subgroup and sensitivity analysis. **a**, Receiver operating characteristic (ROC) curves reporting the area under the curve (AUC) with 95% confidence intervals (CI) of the PACS score after shrinkage applied to all (black; *n* = 134 with 85 having PACS), mild (blue; *n* = 89 with 48 having PACS) and severe (red; *n* = 45 with 37 having PACS) COVID-19 patients of the derivation cohort during primary infection (left) as well as validation cohort (right; *n* = 389, 380 and 9 with 212, 205 and 7 having PACS respectively). ROC curves (middle) reporting the AUC of different disease grades within severe COVID-19 patients of the derivation cohort, namely severe pneumonia, mild, moderate and severe ARDS (*n* = 20, 5, 10 and 10 with 15, 4, 9 and 9 having PACS). Disease severity was followed-up and independent of sampling timepoint. **b**, Sensitivity analysis showing ROC curves, AUC and CI of the derivation cohort using post-COVID-19 syndrome as outcome, defined as symptoms enduring longer than 12 weeks after onset of first COVID-19-related symptoms (left; *n* = 134, with 61 showing post-COVID-19 syndrome) as well as using recovery after six months as outcome in the validation cohort (right; *n* = 389, with 90 not having recovered after six months).

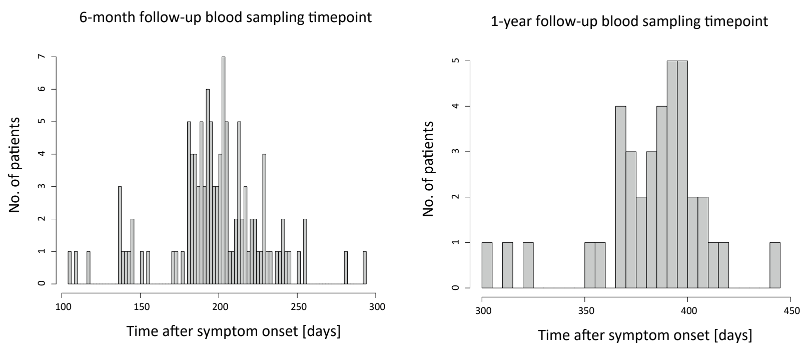

Supplementary Figure 5

Supplementary Fig. 5. Blood sampling timepoints of COVID-19 patient follow-up. Timepoints of blood sampling in COVID-19 patients of derivation cohort in relation to symptom onset. The second visit was planned around six months after symptom onset (left; *n* = 115) with an average timepoint of 199 days (interquartile range 185–216) and the third visit around one year after symptom onset (right; *n* = 38) with an average timepoint of 383 days (interquartile range 371–397).

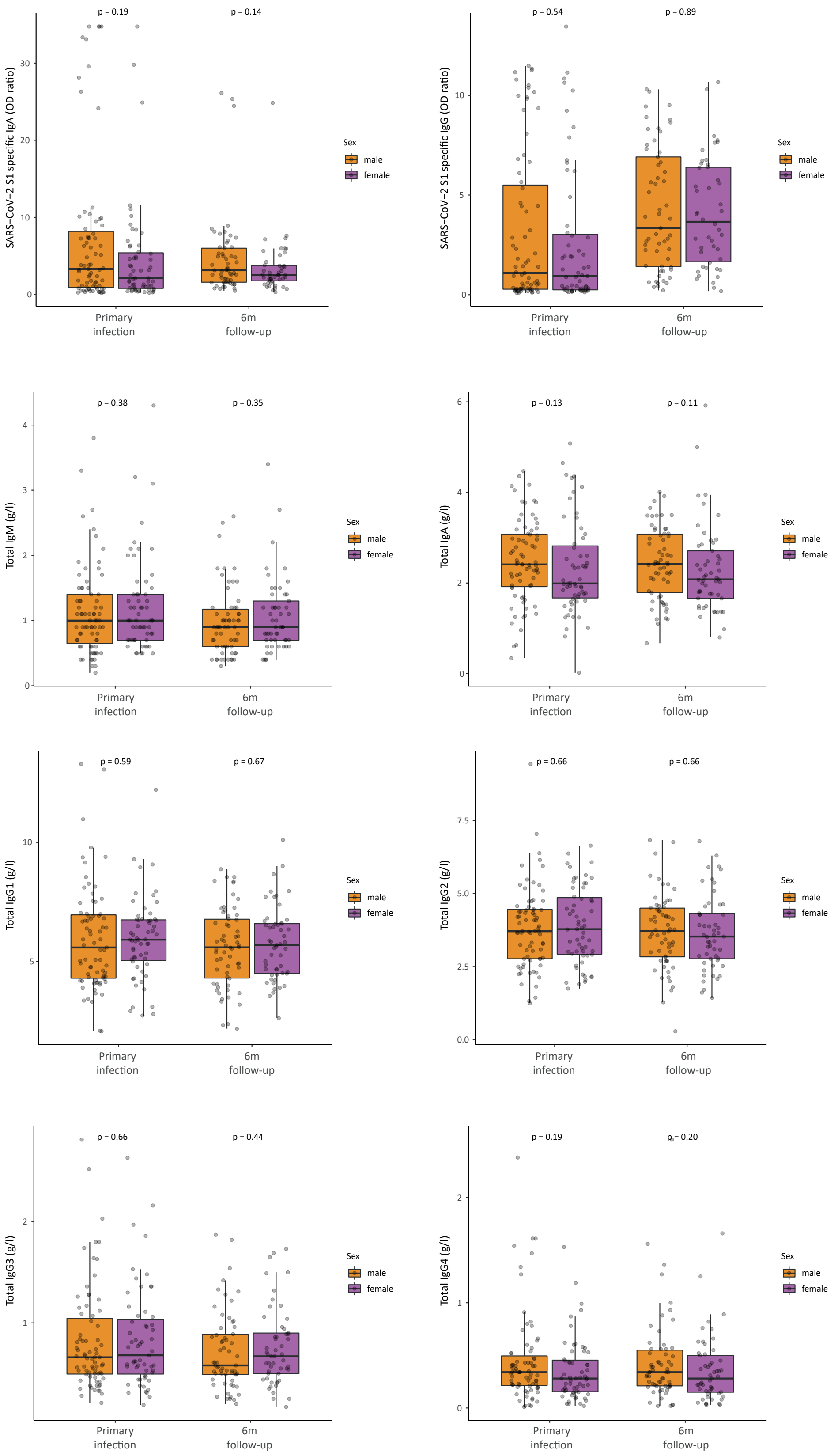

Supplementary Figure 6

Supplementary Fig. 6. Sex differences in COVID-19 patients experiencing PACS. Comparison of SARS-CoV-2 S1-specific IgA and IgG titers and total immunglobulin concentrations of IgM, IgA, and IgG subclasses IgG1 to IgG4 in male and female COVID-19 patients at primary SARS-CoV-2 infection (*n* = 75 and 59, respectively) and 6-month (6m) follow-up (*n* = 62 and 53, respectively). SARS-CoV-2 vaccinated individuals have been excluded from SARS-CoV-2 S1-specific IgA and IgG analysis at 6m follow-up (adjusted *n* = 57 and 46, respectively). Boxplots represent median (middle line) with upper and lower quartiles (box limits), and 1.5*interquartile ranges (whiskers). Variables were compared using two-sided Wilcoxon's test.
