# Supplementary material for: Immunoglobulin signature predicts risk of post-acute COVID-19 syndrome
Source: Nat Commun. 2022 Jan 25;13:446. doi: 10.1038/s41467-021-27797-1 (PMC8789854; doi:10.1038/s41467-021-27797-1)
Supplement: Supplementary file 2 — Description of Additional Supplementary Files [file 41467_2021_27797_MOESM2_ESM.pdf]

Description of Additional Supplementary Files

File Name: Supplementary Software 1
Description: R code for immunoglobulin signature analysis and prediction model development
