# Supplementary material for: Immunoglobulin signature predicts risk of post-acute COVID-19 syndrome
Source: Nat Commun. 2022 Jan 25;13:446. doi: 10.1038/s41467-021-27797-1 (PMC8789854; doi:10.1038/s41467-021-27797-1)
Supplement: Supplementary file 4 — Reporting Summary [file 41467_2021_27797_MOESM4_ESM.pdf]

nature research

Corresponding author(s): Onur Boyman

Last updated by author(s): Dec 8, 2021

Reporting Summary

Nature Research wishes to improve the reproducibility of the work that we publish. This form provides structure for consistency and transparency in reporting. For further information on Nature Research policies, see our Editorial Policies and the Editorial Policy Checklist.

Statistics

For all statistical analyses, confirm that the following items are present in the figure legend, table legend, main text, or Methods section.

n/a	Confirmed	
☐	☒	The exact sample size (*n*) for each experimental group/condition, given as a discrete number and unit of measurement
☐	☒	A statement on whether measurements were taken from distinct samples or whether the same sample was measured repeatedly
☐	☒	The statistical test(s) used AND whether they are one- or two-sided<br>*Only common tests should be described solely by name; describe more complex techniques in the Methods section.*
☐	☒	A description of all covariates tested
☐	☒	A description of any assumptions or corrections, such as tests of normality and adjustment for multiple comparisons
☐	☒	A full description of the statistical parameters including central tendency (e.g. means) or other basic estimates (e.g. regression coefficient) AND variation (e.g. standard deviation) or associated estimates of uncertainty (e.g. confidence intervals)
☐	☒	For null hypothesis testing, the test statistic (e.g. *F*, *t*, *r*) with confidence intervals, effect sizes, degrees of freedom and *P* value noted<br>*Give P values as exact values whenever suitable.*
☒	☐	For Bayesian analysis, information on the choice of priors and Markov chain Monte Carlo settings
☐	☒	For hierarchical and complex designs, identification of the appropriate level for tests and full reporting of outcomes
☒	☐	Estimates of effect sizes (e.g. Cohen's *d*, Pearson's *r*), indicating how they were calculated

Our web collection on statistics for biologists contains articles on many of the points above.

Software and code

Policy information about availability of computer code

Data collection	Microsoft Office Excel (version 2102)
Data analysis	Statistical analyses were performed with R (version 4.1.2) and using the packages "biostatUZH" (version 1.8.0), "CalibrationCurves" (version 0.1.2), "dcurves" (version 0.2.0), "epitools" (version 0.5-10.1), "interactions" (version 1.1.5), "gbm" (version 2.1.8), "ggstatsplot" (version 0.9.0), "interactions", "pROC" (version 1.18.0), "mgcv" (version 1.8-38), "shrink" (version 1.2.1), and "sjPlot" (version 2.8.9).<br>R code is provided in the Supplementary Information.

For manuscripts utilizing custom algorithms or software that are central to the research but not yet described in published literature, software must be made available to editors and reviewers. We strongly encourage code deposition in a community repository (e.g. GitHub). See the Nature Research guidelines for submitting code & software for further information.

Data

Policy information about availability of data

All manuscripts must include a data availability statement. This statement should provide the following information, where applicable:
- Accession codes, unique identifiers, or web links for publicly available datasets
- A list of figures that have associated raw data
- A description of any restrictions on data availability

All relevant data generated in this study are provided in the Supplementary Information. A PACS score calculator will be accessible online.

Field-specific reporting

Please select the one below that is the best fit for your research. If you are not sure, read the appropriate sections before making your selection.

- [x] Life sciences
- [ ] Behavioural & social sciences
- [ ] Ecological, evolutionary & environmental sciences

For a reference copy of the document with all sections, see nature.com/documents/nr-reporting-summary-flat.pdf

Life sciences study design

All studies must disclose on these points even when the disclosure is negative.

Sample size	215 adult individuals were included in the derivation cohort of the study, of which 157 were followed-up. The sample size for the development of our prediction model was obtained by including and following up all consecutive patients between April 2020 and August 2021 and resulted in a total of 134 followed-up patients. The prediction model (PACS score) was developed using 14.2 events per predictor parameter, which is in line with the rule of thumb of 15 events per predictor parameter as well as several other recommendations on the required number of events per predictor parameter for accurate modelling in logistic regression analysis.<br>395 adult individuals were included as a validation cohort, between 06 August 2020 and 19 January 2021 and prospectively followed-up for six months after infection, which is in line with a suggested sample size of 400 and an outcome event size of 200 in order to obtain precise calibration curves (see Methods).
Data exclusions	No data was excluded.
Replication	Samples from each patient were analyzed once due to limited availability. As prognostic models tend to describe optimally the evaluated dataset, but may perform less well in other datasets, we addressed this phenomenon of overfitting by applying the statistical method of shrinkage (see Methods). The prognostic model was validated in an independent validation cohort.
Randomization	This is not relevant for this observational study.
Blinding	While performing measurements the investigators were fully blinded. Blinding was not possible for data analysis as the study was observational.

Reporting for specific materials, systems and methods

We require information from authors about some types of materials, experimental systems and methods used in many studies. Here, indicate whether each material, system or method listed is relevant to your study. If you are not sure if a list item applies to your research, read the appropriate section before selecting a response.

Materials & experimental systems

n/a	Involved in the study	
[ ]	[x]	Antibodies
[x]	[ ]	Eukaryotic cell lines
[x]	[ ]	Palaeontology and archaeology
[x]	[ ]	Animals and other organisms
[ ]	[x]	Human research participants
[x]	[ ]	Clinical data
[x]	[ ]	Dual use research of concern

Methods

n/a	Involved in the study	
[x]	[ ]	ChIP-seq
[x]	[ ]	Flow cytometry
[x]	[ ]	MRI-based neuroimaging

Antibodies

Antibodies used	All laboratory tests were performed in accredited laboratories at the University Hospital Zurich. Blood samples were collected in BD Vacutainer CAT serum tubes (Becton Dickinson; Cat# 367896). Different serum immunoglobulins subsets and IgG subclasses were measured using the commercially available turbidimetric Optilite® assays using an Optilite® analyzer (The Binding Site Group Ltd; Cat# NK004.OPT, NK006–NK010.OPT, NK012.OPT). Laboratory reference values are as follows (g/l): IgM (0.4–2.8), IgA (0.7–4.0), IgG (7.0–16.0), IgG1 (2.8–8.0), IgG2 (1.15–5.70), IgG3 (0.24–1.25), IgG4 (0.052–1.25). SARS-CoV-2-specific IgA and IgG antibodies were measured by using a commercial enzyme-linked immunosorbent assay (ELISA) specific for the SARS-CoV-2 spike S1 protein (Euroimmun SARS-CoV-2 IgA and IgG immunoassay; Cat# EI 2606-9601). Interleukin IL-6 and tumor necrosis factor (TNF) were quantified using R&D Systems assays (Cat# S6050 and LHSCM210, respectively). Antibody dilutions were prepared according to the manufacturer's instructions.
Validation	All laboratory tests were performed in accredited laboratories at the University Hospital Zurich. All antibodies used in this study are part of commercially available assays. Primary antibodies:<br>SARS-CoV-2-specific IgA and IgG (Euroimmun; Cat# EI 2606-9601 A and G). Specificity: SARS-CoV-2 spike S1 domain (Wuhan-Hu-1). Cross-reactivity: < 0.5% cross-reactivity observed with other human coronaviruses.<br>Human IL-6 Quantikine ELISA Kit (R&D Systems, Cat# S6050 Specificity: Natural and recombinant human IL-6. Cross-reactivity: < 0.5%

cross-reactivity observed with available related molecules.
Human TNF-alpha High Sensitivity Magnetic Luminex Performance Assay (R&D Systems, Cat# LHSCM210). Specificity: Natural and recombinant human TNF-alpha. Validated for use with serum and plasma samples (Sensitivity, intra-assay precision, inter-assay precision, linearity and recovery), as described on the manufacturer's website.

Human research participants

Policy information about studies involving human research participants

Population characteristics	Participant characteristics are shown in Table 1.
Recruitment	Adult individuals were included and followed-up in the study between April 2020 and August 2021. The recruitment is described in the methods section. A flow chart of the study design is shown in Figure 1. Symptomatic patients with RT-qPCR-confirmed SARS-CoV-2 infection were recruited at four different hospitals in Zurich, Switzerland. Both outpatients and hospitalized patients were recruited into the study. All participants gave written informed consent and had to be competent at the time of consent. Thus, patients that were already hospitalized at inclusion time point must have been able to give informed consent, which may have introduced a potential bias for patients who were mechanically ventilated at the time of hospitalization and could therefore be only recruited before or afterwards.
Ethics oversight	The study was approved by the Cantonal Ethics Committee of Zurich (BASEC #2016-01440) and all participants were included following written informed consent.

Note that full information on the approval of the study protocol must also be provided in the manuscript.
